# Supplementary material for: Fed the same way? Exploring the influence of breastfeeding, weaning, and childhood diet on adult sex ratios through stable isotope analysis of incremental dentine in Medieval Tuscany, Italy (11th–15th c. CE)
Source: PLoS One. 2026 Jan 21;21(1):e0338595. doi: 10.1371/journal.pone.0338595 (PMC12822958; doi:10.1371/journal.pone.0338595)

**Fed the Same Way? Exploring the Influence of Breastfeeding, Weaning, and Childhood Diet on Adult Sex Ratios Through Stable Isotope Analysis of Incremental Dentine in Medieval Tuscany, Italy (11^th^ – 15^th^ c. CE)**

Alessio Amaro^1*^, Antonio Fornaciari^2^, Valentina Giuffra^2^, Sang-Tae Kim^3^, Martin Knyf^3^, Paul Szpak^4^, Bonnie Kahlon^1^, Tracy L. Prowse^1^

^1^Department of Anthropology, McMaster University, Canada

^2^Department of Translational Research and New Technologies in Medicine and Surgery, University of Pisa, Italy

^3^School of Geography and Earth Sciences, McMaster University, Canada

^4^Department of Anthropology, Trent University, Canada

*Corresponding Author: amaroa@mcmaster.ca

| **ID** | **Chronology** | **Biological sex** | **Age-at-death** |
| --- | --- | --- | --- |
| ***Aulla*** | | | |
| AU1660 | 12^th^ - 13^th^ | Male | 25-30 |
| AU1684 | 13^th^ - 14^th^ | Male | 25-35 |
| AU1685 | 12^th^ - 13^th^ | Male | 25-30 |
| AUT52 | 11^th^ | Male | 30+ |
| AUT39 | 11^th^ - 13^th^ | Female | 30+ |
| AUT4 | 13^th^ - 14^th^ | Male | 30+ |
| AUT3 | 13^th^ - 14^th^ | Female | 30+ |
| AUT22 | 11^th^ - 12^th^ | Female | 30+ |
| AU1636 | 13^th^ - 14^th^ | Male | 30-35 |
| AU1637 | 13^th^ - 14^th^ | Male | 30-35 |
| AUT15 | 13^th^ - 14^th^ | Female | 30+ |
| ***Badia Pozzeveri*** | | | |
| BP6015 | 12^th^ - 13^th^ | Male | 20-25 |
| BP3641 | 11^th^ - 12^th^ | Male | 20-25 |
| BP3746 | 11^th^ - 12^th^ | Male | 30-40 |
| BP3640 | 11^th^ - 12^th^ | Male | 20-25 |
| BP2730 | 11^th^ - 12^th^ | Male | 35-40 |
| BP3743 | 11^th^ - 12^th^ | Female | 40-55 |
| BP6065 | 12^th^ - 13^th^ | Female | 40-55 |
| BP2776 | 11^th^ - 12^th^ | Male | 45-55 |
| BP3653 | 11^th^ - 12^th^ | Female | 25-35 |
| BP6182 | 12^th^ - 13^th^ | Male | 30-40 |
| BP3855 | 11^th^ - 12^th^ | Female | 25-35 |
| BP6053 | 12^th^ - 13^th^ | Male | 35-40 |
| BP3781 | 11^th^ - 12^th^ | Female | 35-45 |
| BP6130 | 12^th^ - 13^th^ | Male | 25-30 |
| BP6175 | 12^th^ - 13^th^ | Male | 30-45 |
| BP6165 | 12^th^ - 13^th^ | Male | 25-30 |
| BP3730 | 11^th^ - 12^th^ | Male | 30-40 |
| BP6165 | 12^th^ - 13^th^ | Male | 25-30 |
| BP6082 | 12^th^ - 13^th^ | Male | 20-25 |
| BP6071 | 12^th^ - 13^th^ | Male | 20-25 |
| BP6054 | 12^th^ - 13^th^ | Male | 40-50 |
| BP3588 | 11^th^ - 12^th^ | Female | 45-55 |
| ***Montescudaio*** | | | |
| MTSCT9US40254 | 15^th^ | Male | 35-50 |
| MTSC210 | 12^th^ - 14^th^ | Female | 25-30 |
| MTSC3 ALT IND | 15^th^ | Female | 50+ |
| MTSC34 | 12^th^ - 14^th^ | Female | 25-30 |
| MTSC63 | 12^th^ - 14^th^ | Female | 35-40 |
| MTSC2936 | 12^th^ - 14^th^ | Male | 20-25 |
| MTSCT9US40251 | 15^th^ | Male | 30+ |
| MTSC452 | 11^th^ | Male | 20-25 |
| MTSCT49 US 1792 | 12^th^ - 14^th^ | Male | 30+ |
| MTSC3857 | 12^th^ - 14^th^ | Male | 30+ |
| MTSC56 | 12^th^ - 14^th^ | Male | 20-30 |
| MTSC444 | 11^th^ | Female | 35-40 |
| MTSC482 | 11^th^ | Female | 25-30 |
| MTSC503 | 12^th^ - 14^th^ | Female | 30-35 |
| MTSC9 | 15^th^ | Male | 30+ |
| MTSC402 | 12^th^ - 14^th^ | Male | 20-25 |
| MTSC297 | 12^th^ - 14^th^ | Female | 25-30 |
| MTSC46 | 12^th^ - 14^th^ | Male | 20-25 |
| MTSC371 | 11^th^ | Male | 20-25 |
| MTSC2 | 15^th^ | Female | 30+ |
| MTSC474 | 11^th^ | Male | 25-30 |
| MTSC368 | 12^th^ - 14^th^ | Female | 25-30 |
| MTSC501 | 11^th^ | Female | 30-35 |
| MTSC73 | 12^th^ - 14^th^ | Male | 45-50 |
| MTSC158 | 11^th^ | Male | 30-35 |
| MTSC507 | 11^th^ | Male | 20-25 |
| MTSC472 | 11^th^ | Male | 20-25 |
| MTSC298 US 4704 | 12^th^ - 14^th^ | Female | 40-50 |
| MTSC483 | 11^th^ | Male | 30+ |
| MTSC75 | 12^th^ - 14^th^ | Male | 20-25 |
| MTSC505 | 11^th^ | Female | 35-40 |

Table 1. List of individuals divided by site with demographic information

| First dentine sections | n= number of dentine sections | δ^13^C (‰) VPDB (S.D.) | δ^15^N (‰) AIR (S.D) | n= number of dentine sections | δ^13^C (‰) VPDB (S.D.) | δ^15^N (‰) AIR (S.D.) |
| --- | --- | --- | --- | --- | --- | --- |
| *Aulla phase 1 (11^th^-13^th^ c. CE)* | ***Males*** | | | ***Females*** | | |
| ~ 9 m. | 3 | -19.2 ± 1.3 | 10.4 ± 2.0 | 1 | -19.0 | 10.8 |
| *Aulla phase 2 (13^th^-14^th^ c. CE)* | ***Males*** | | | ***Females*** | | |
| ~ 9 m. | 2 | -17.2 ± 1.2 | 11.3 ± 0.7 | 1 | -17.8 | 12.6 |

Table 2. Mean *δ*^13^C and *δ*^15^N for each first section at Aulla.

| First dentine sections | n= number of dentine sections | δ^13^C (‰) VPDB (S.D.) | δ^15^N (‰) AIR (S.D) | n= number of dentine sections | δ^13^C (‰) VPDB (S.D.) | δ^15^N (‰) AIR (S.D.) |
| --- | --- | --- | --- | --- | --- | --- |
| *Badia Pozzeveri phase 1 (11^th^-12^th^ c. CE)* | ***Males*** | | | ***Females*** | | |
| ~ 9 m. | 4 | -17.0 ± 0.7 | 11.6 ± 1.4 | 4 | -18.0 ± 1.2 | 11.0 ± 1.9 |
| *Badia Pozzeveri phase 2 (12^th^-13^th^ c. CE)* | ***Males*** | | | ***Females*** | | |
| ~ 9 m. | 8 | -16.8 ± 2.3 | 10.6 ± 1.4 | 1 | -15.5 | 13.1 |

Table 3. Mean *δ*^13^C and *δ*^15^N for each first section at Badia Pozzeveri.

| First dentine sections | n= number of dentine sections | δ^13^C (‰) VPDB (S.D.) | δ^15^N (‰) AIR (S.D) | n= number of dentine sections | δ^13^C (‰) VPDB (S.D.) | δ^15^N (‰) AIR (S.D.) |
| --- | --- | --- | --- | --- | --- | --- |
| *Montescudaio phase 1 (11^th^ c. CE)* | ***Males*** | | | ***Females*** | | |
| ~ 9 m. | 7 | -19.0 ± 0.9 | 11.2 ± 1.4 | 3 | -17.9 ± 1.6 | 11.8 ± 1.4 |
| *Montescudaio phase 2 (12^th^-14^th^ c. CE)* | ***Males*** | | | ***Females*** | | |
| ~ 9 m. | 8 | -18.4 ± 1.8 | 11.7 ±2.1 | 6 | -18.2 ± 1.7 | 12.0 ± 2.1 |
| *Montescudaio phase 3 (15^th^ c. CE)* | ***Males*** | | | ***Females*** | | |
| ~ 9 m. | 3 | -19.3 ± 0.1 | 11.9 ± 0.4 | 2 | -18.7 ± 0.5 | 12.1 ± 1.9 |

Table 4. Mean *δ*^13^C and *δ*^15^N for each first section at Montescudaio.


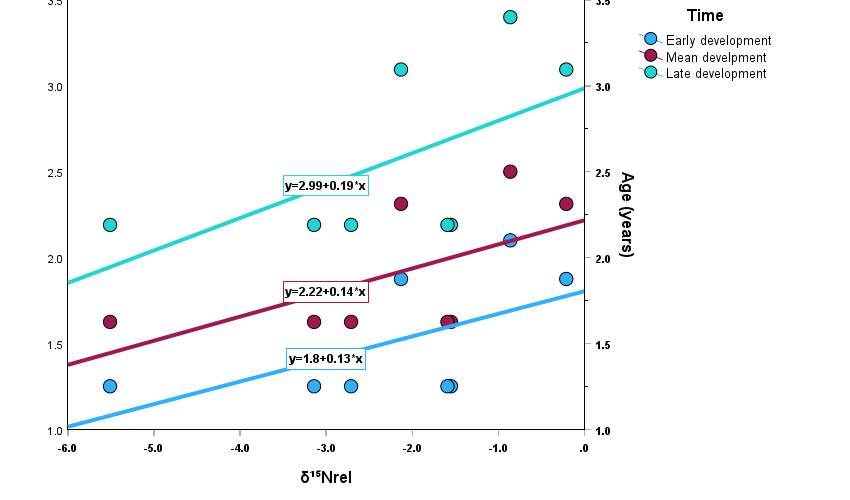


Fig 1. Aulla weaning end (males and females combined). The intersection of the trend lines and the y-axis represent the age at which weaning ended based on early, mean and late development of first permanent molars.


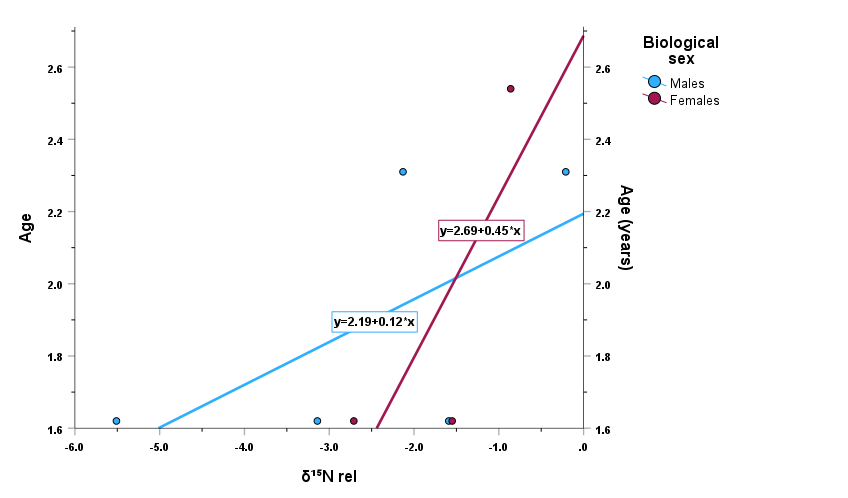


Fig 2. Estimate of weaning end for males (blue) and females (red) at Aulla. The intersection of the trend line and the y-axis represents the age at which weaning ended.


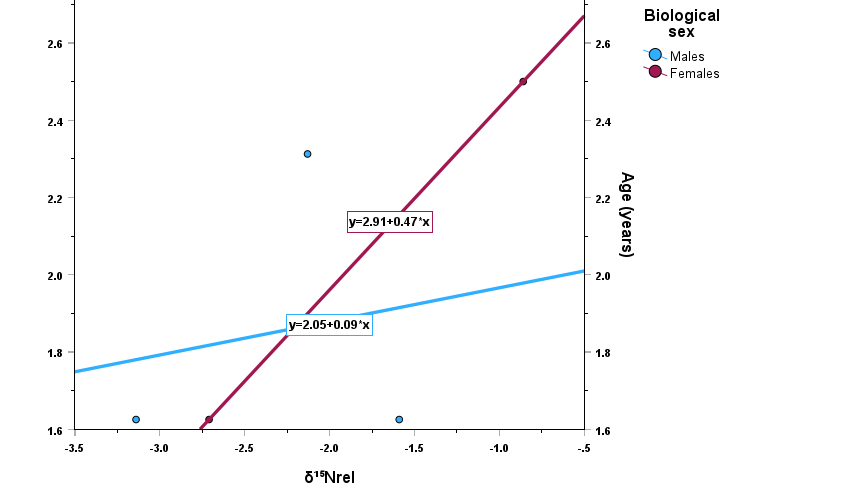


Fig 3. Estimate of weaning end for males (blue) and females (red) at Aulla during phase 2 (13^th^-14^th^ c. CE). The intersection of the trend line and the y-axis represents the age at which weaning ended.


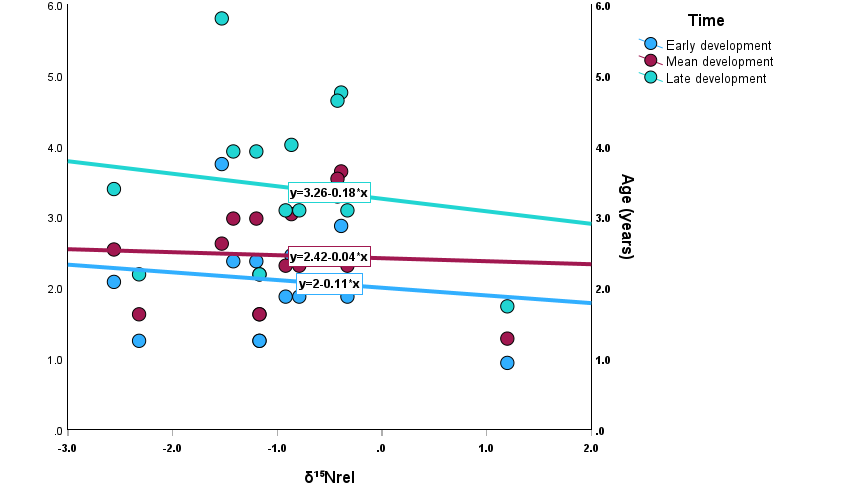


Fig 4. Estimate of weaning end at Badia Pozzeveri (males and females combined). The intersection of the trend lines and the y-axis represent the age at which weaning ended based on early, mean and late development of first permanent molars.


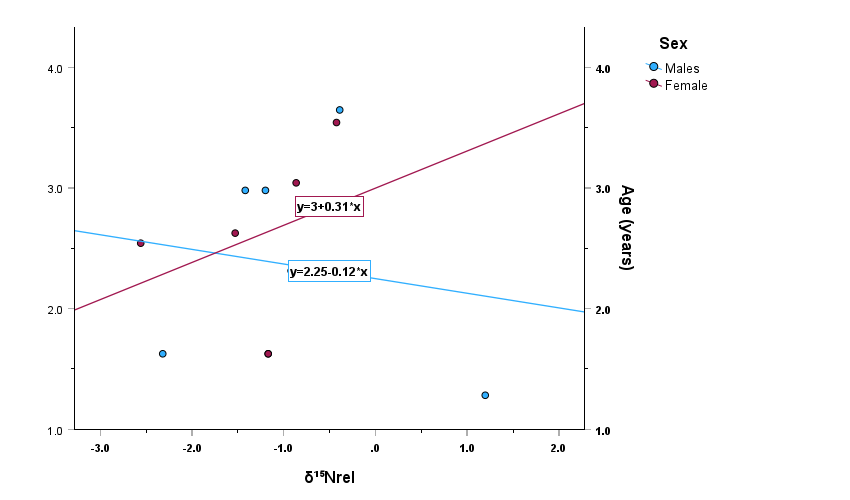


Fig 5. Estimate of weaning end for males (blue) and females (red) at Badia Pozzeveri. The intersection of the trend line and the y-axis represents the age at which weaning ended.


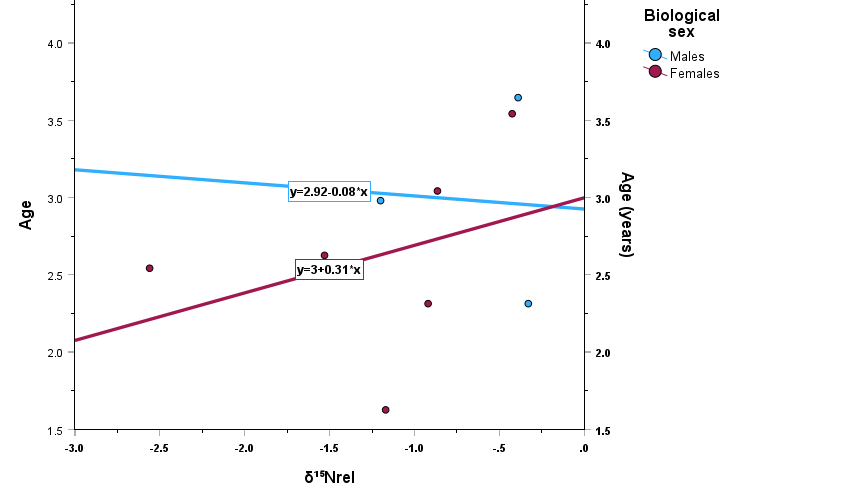


Fig 6. Estimate of weaning end for males (blue) and females (red) at Badia Pozzeveri during phase 1 (11^th^-12^th^ c. CE). The intersection of the trend line and the y-axis represents the age at which weaning ended.


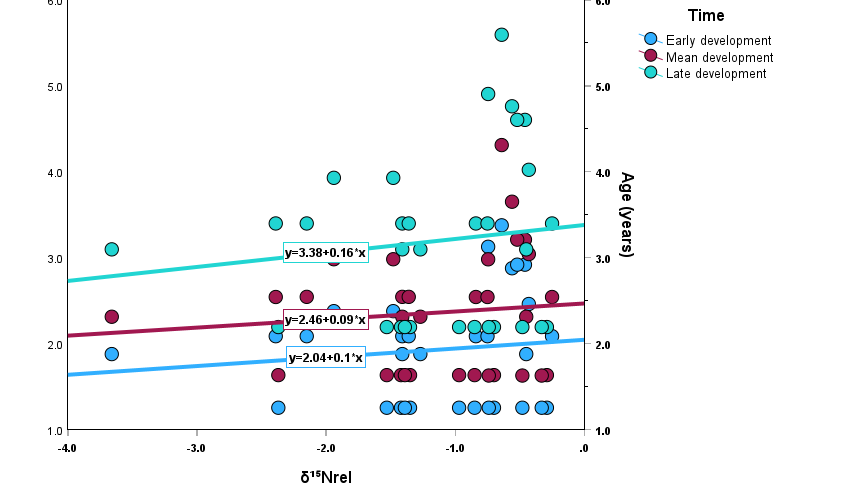


Fig 7. Estimate of weaning end (males and females combined) at Montescudaio. The intersection of the trend lines and the y-axis represent the age at which weaning ended based on early, mean and late development of first permanent molars.


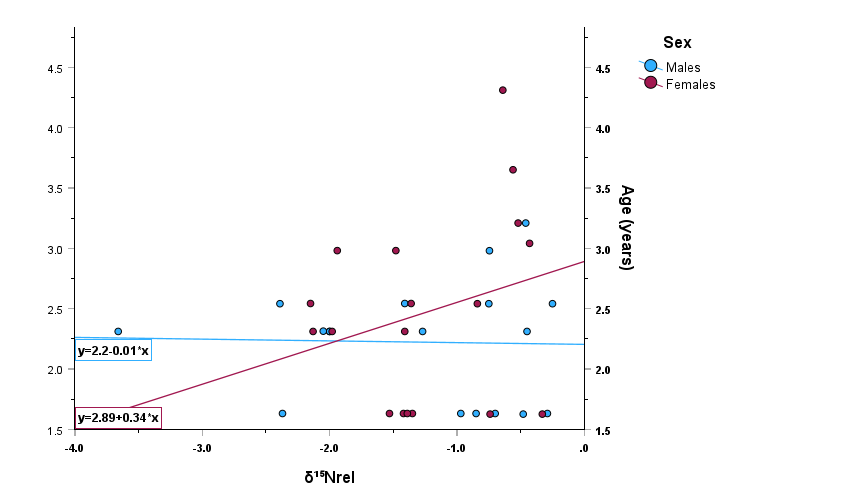


Fig 8. Estimate of weaning end for males (blue) and females (red) at Montescudaio. The intersection of the trend line and the y-axis represents the age at which weaning ended.


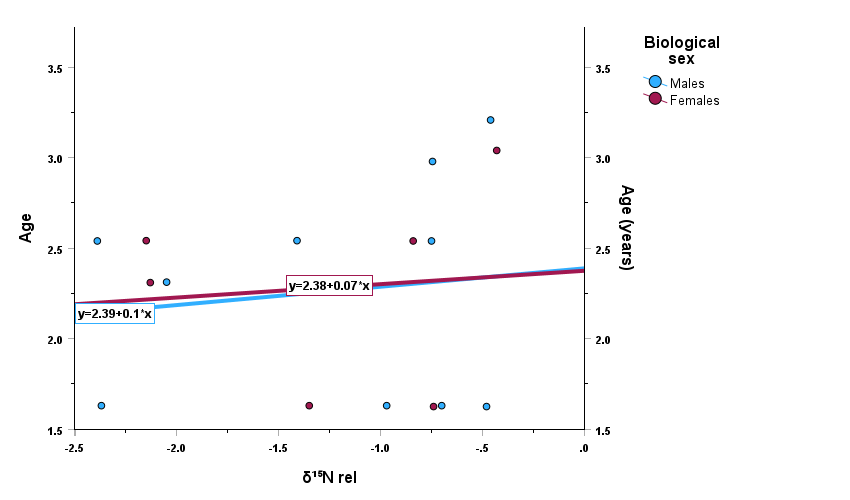


Fig 9. Estimate of weaning end for males (blue) and females (red) at Montescudaio during phase 1 (11^th^ c. CE) (males and females). The intersection of the trend line and the y-axis represents the age at which weaning ended.


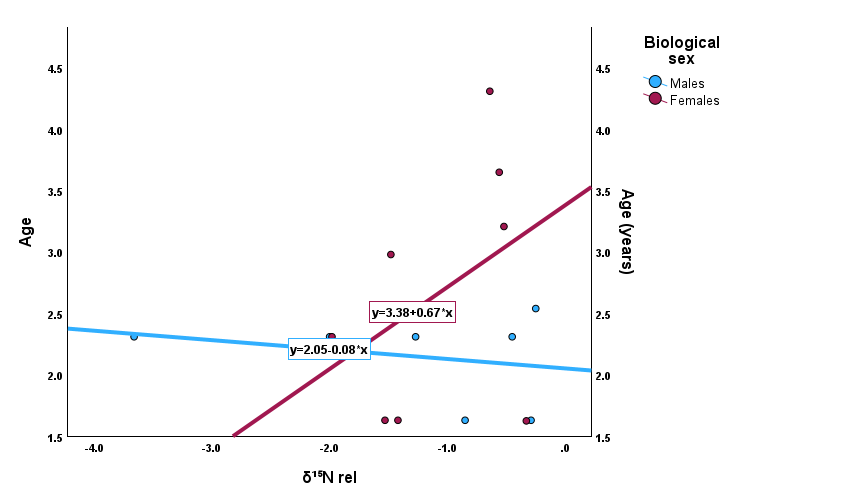


Fig 10. Estimate of weaning end for males (blue) and females (red) at Montescudaio during phase 2 (12^th^ – 14^th^ c. CE). The intersection of the trend line and the y-axis represents the age at which weaning ended.

***Aulla δ*^13^C and *δ*^15^N profiles**


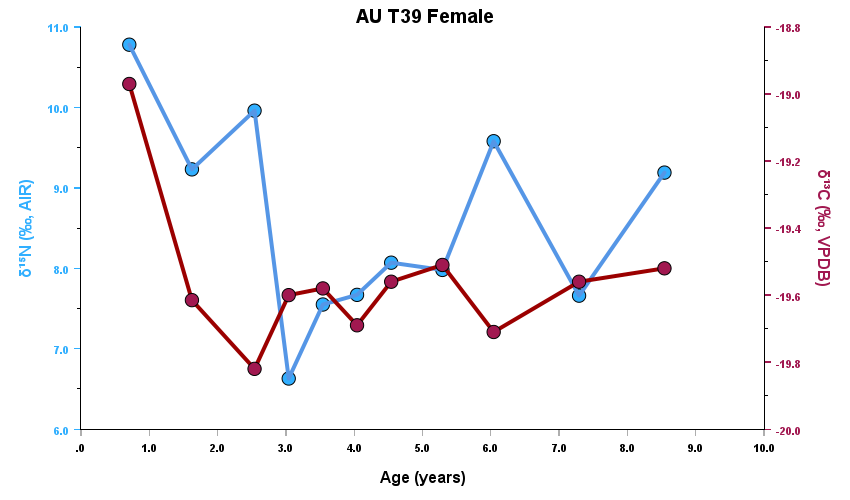

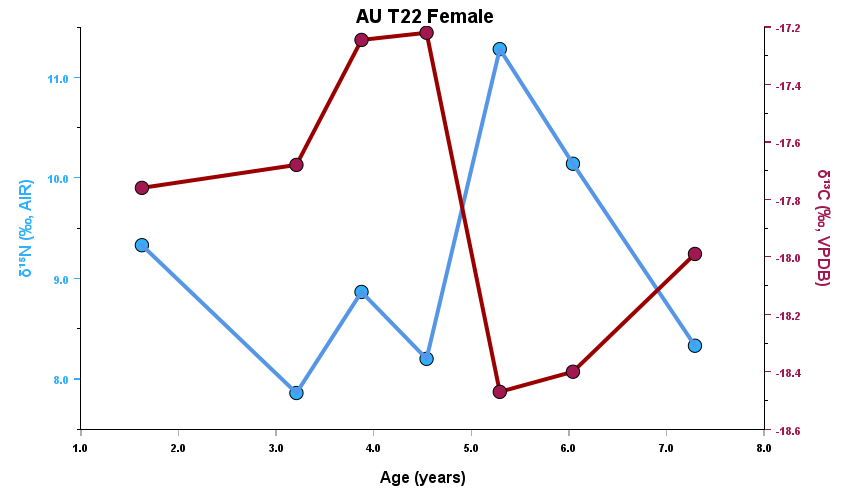

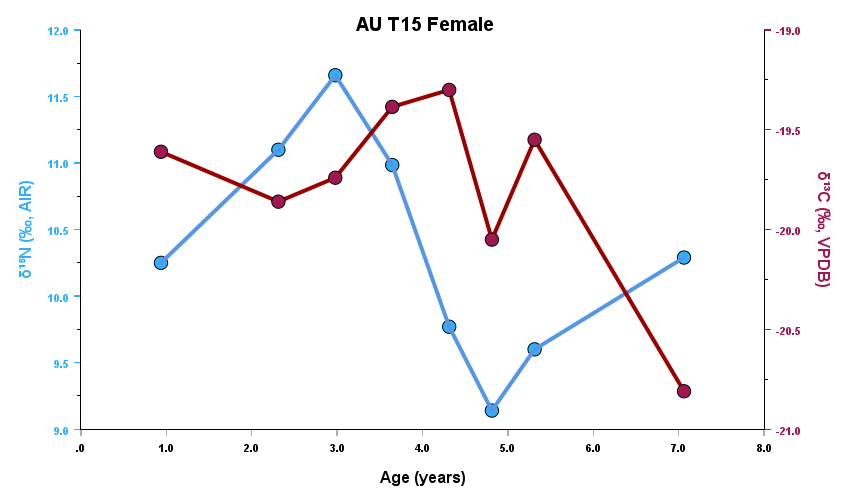

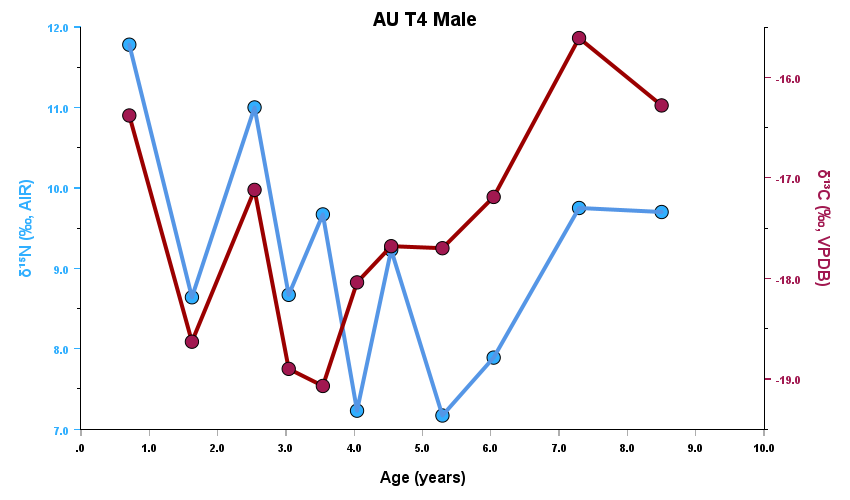

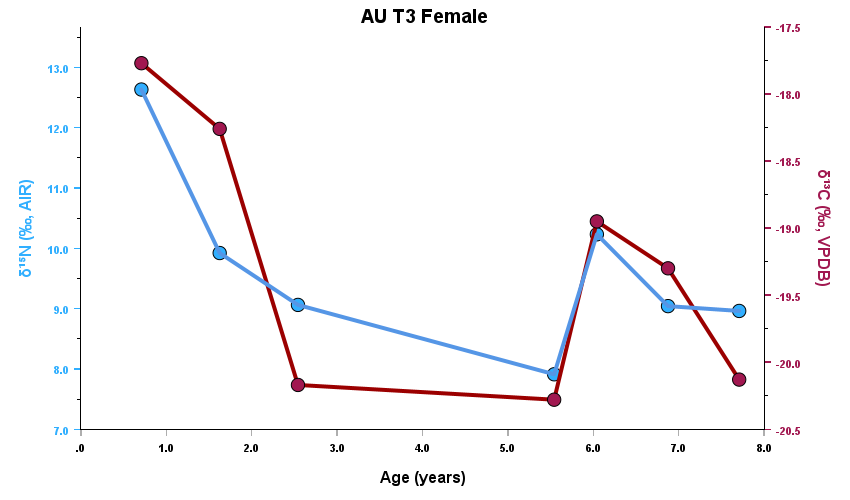

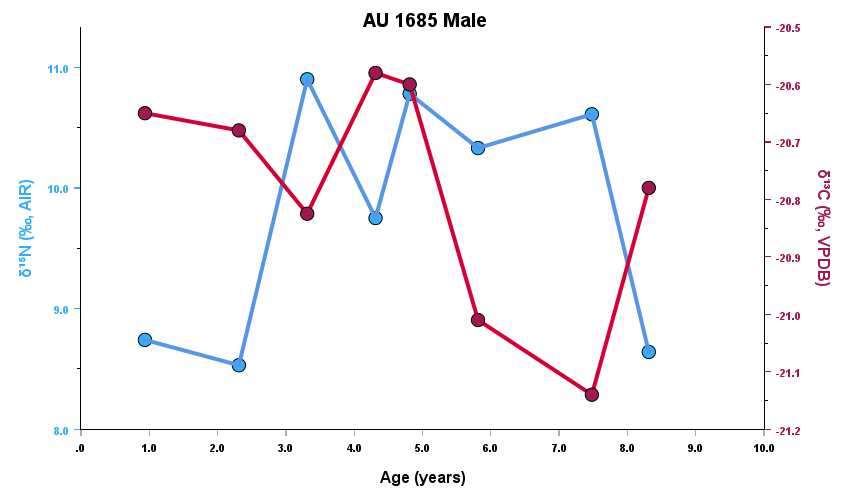

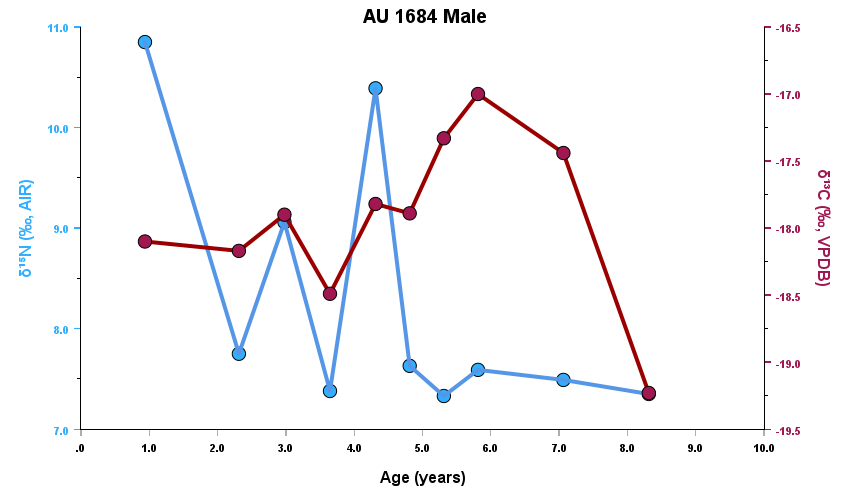

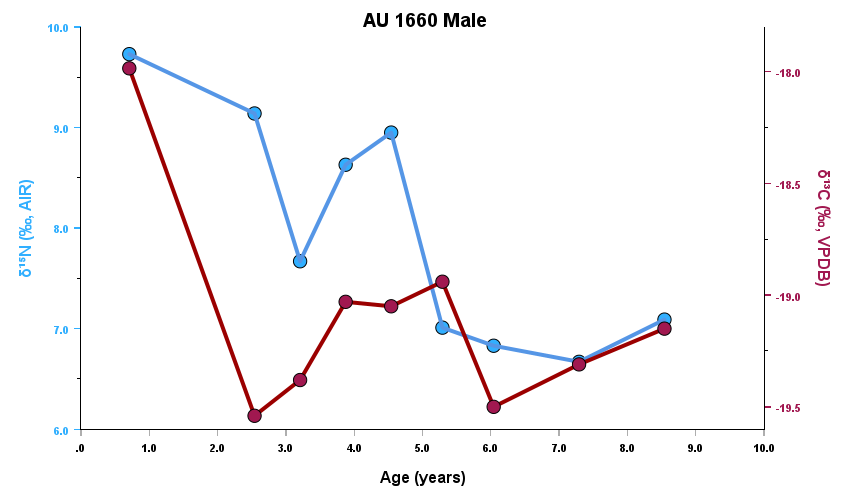

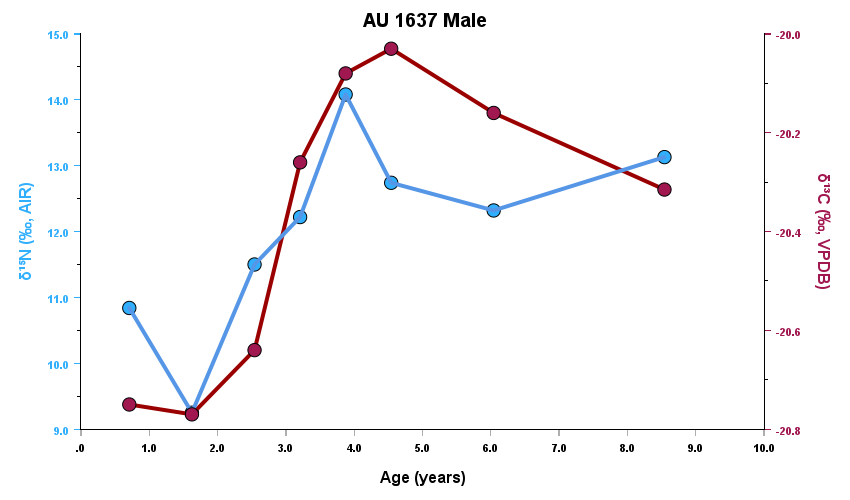

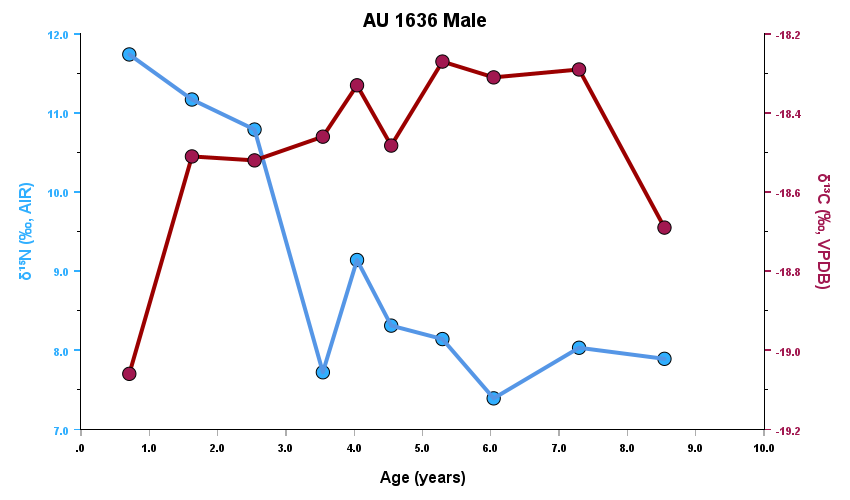


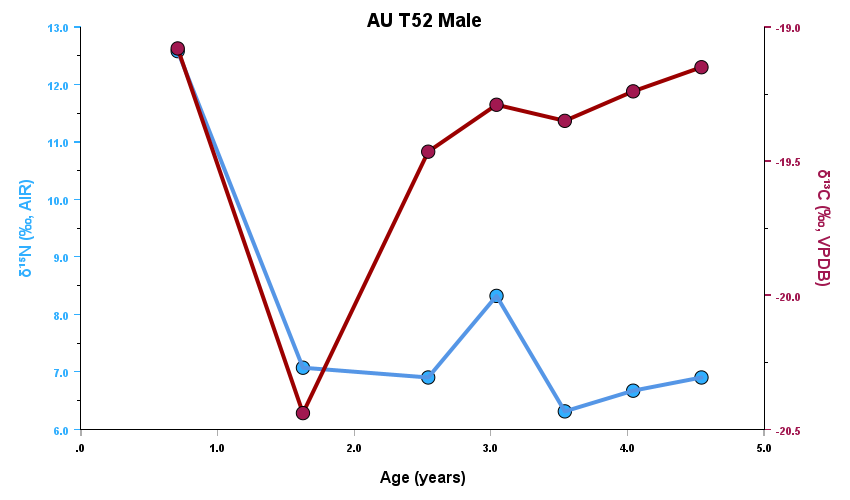


***Badia Pozzeveri δ*^13^C and *δ*^15^N profiles**


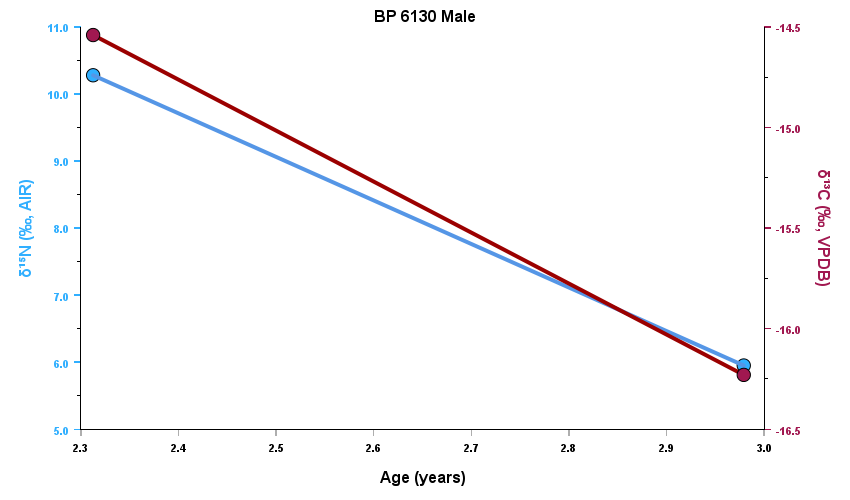

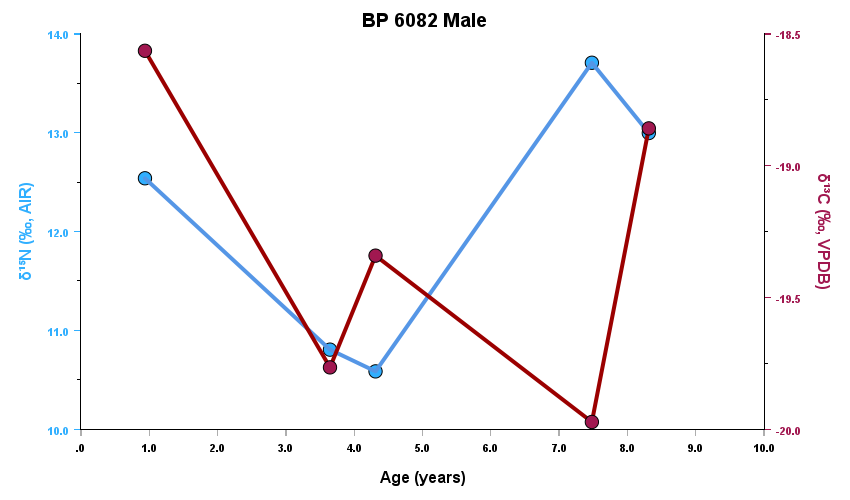

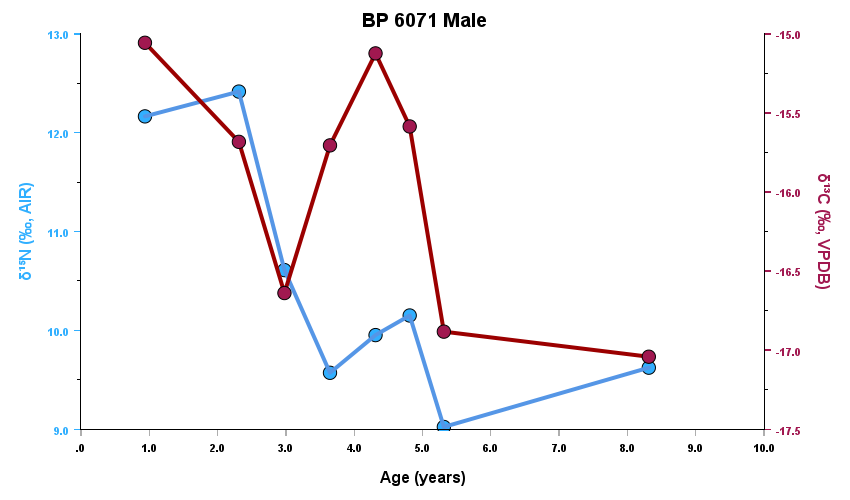

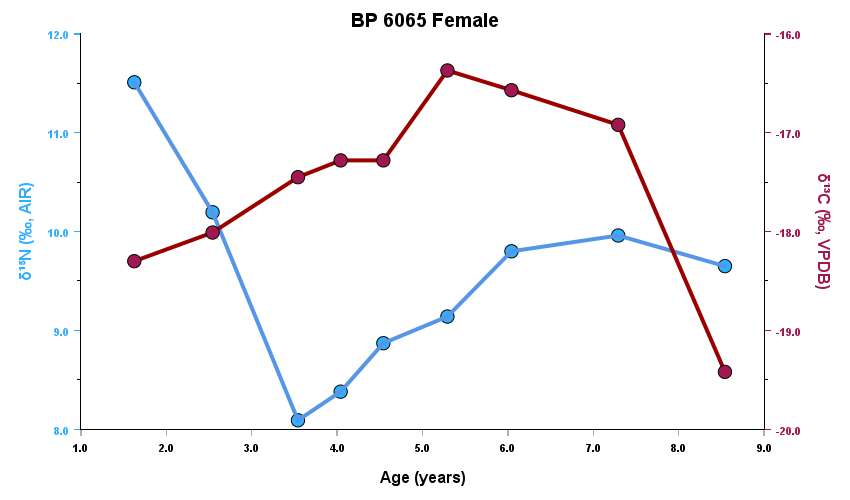

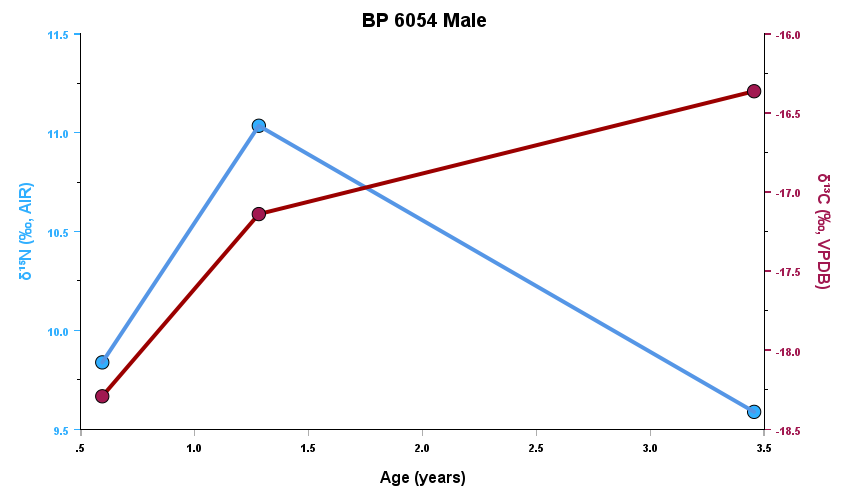

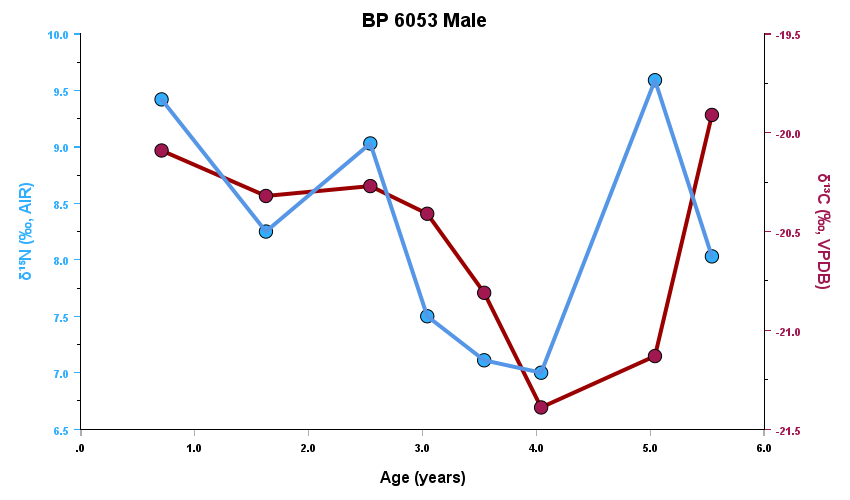

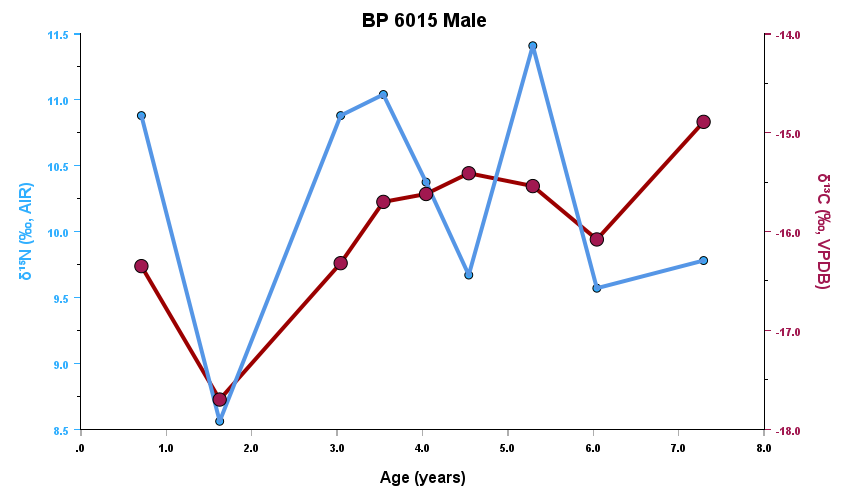

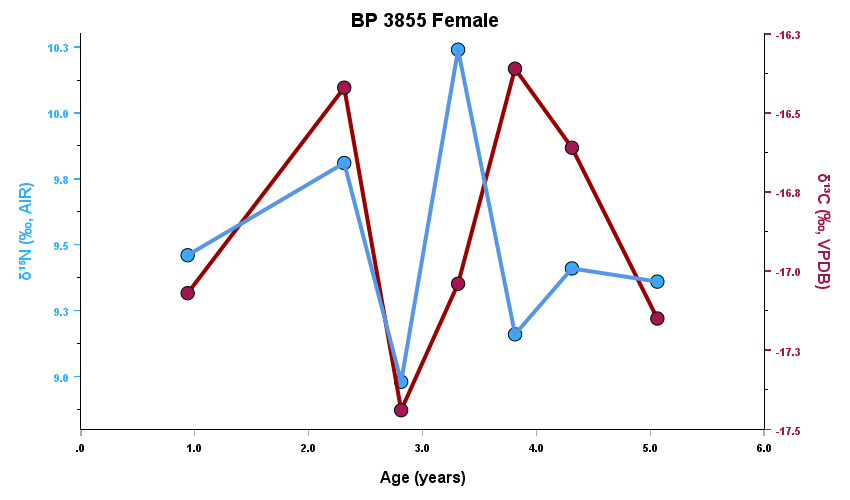

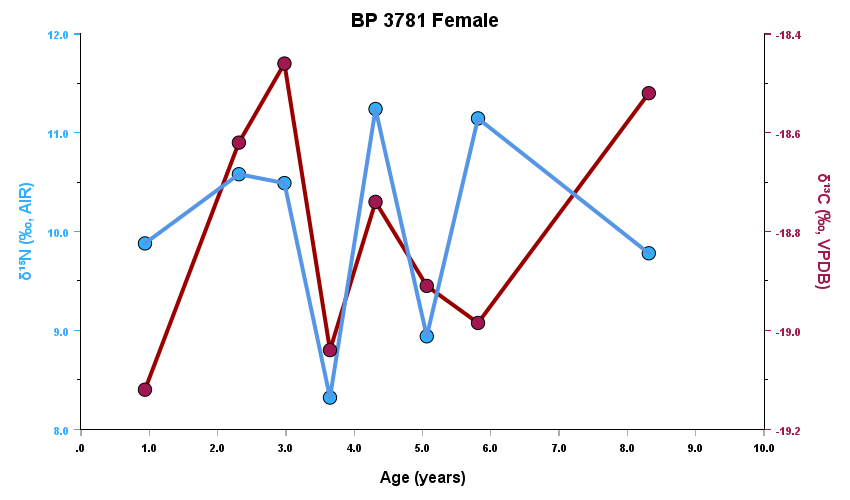

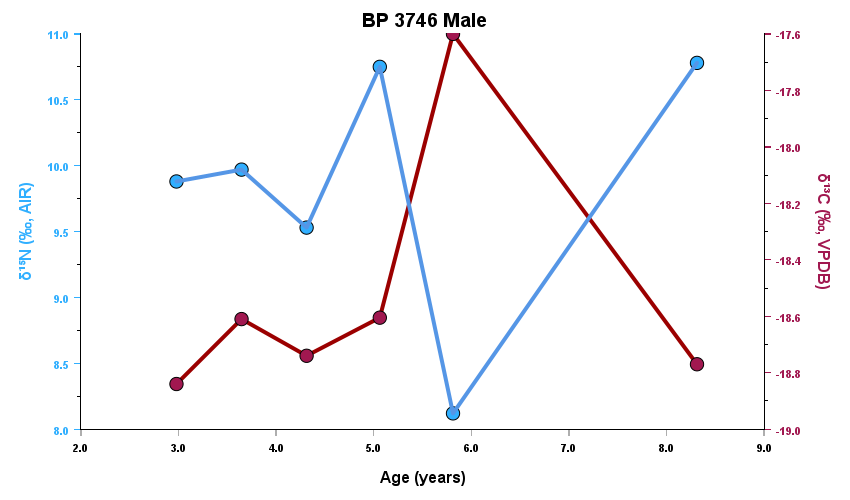

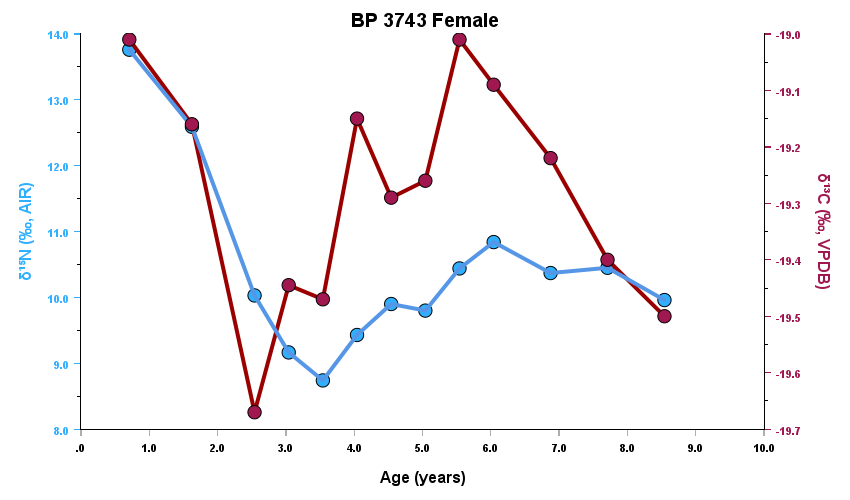

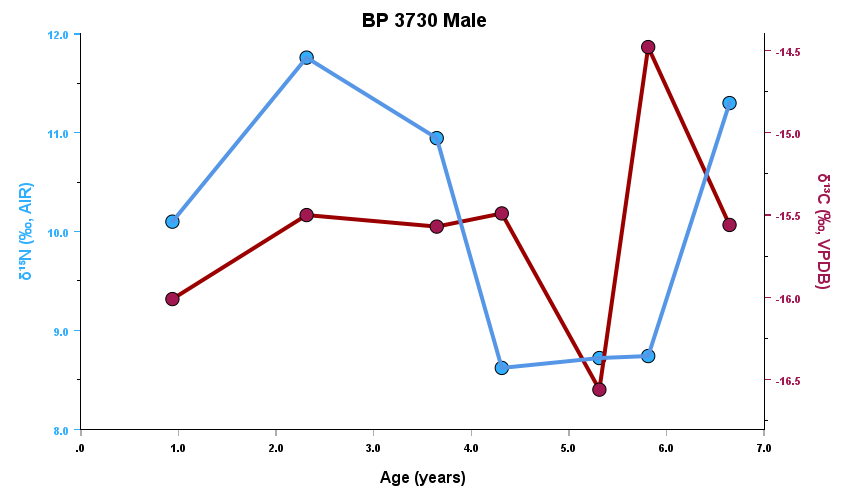

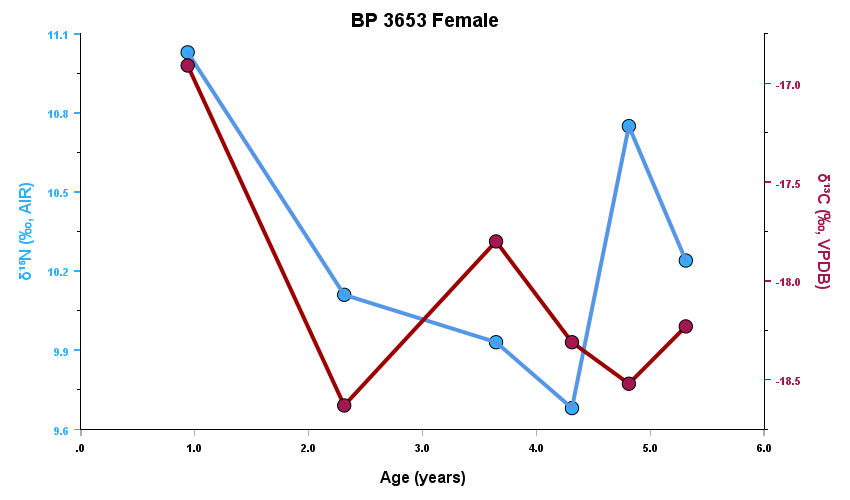

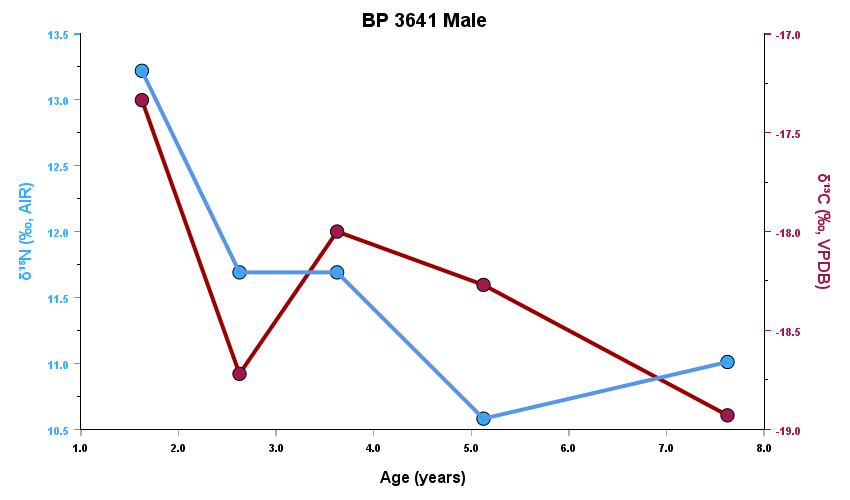

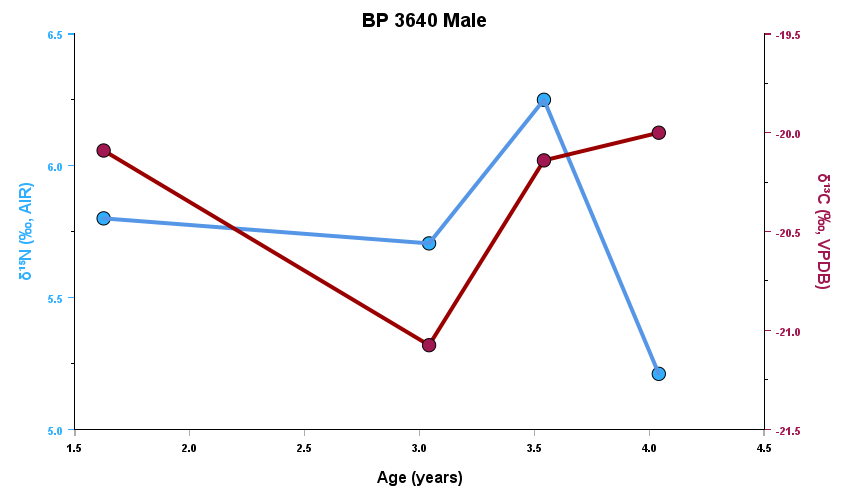

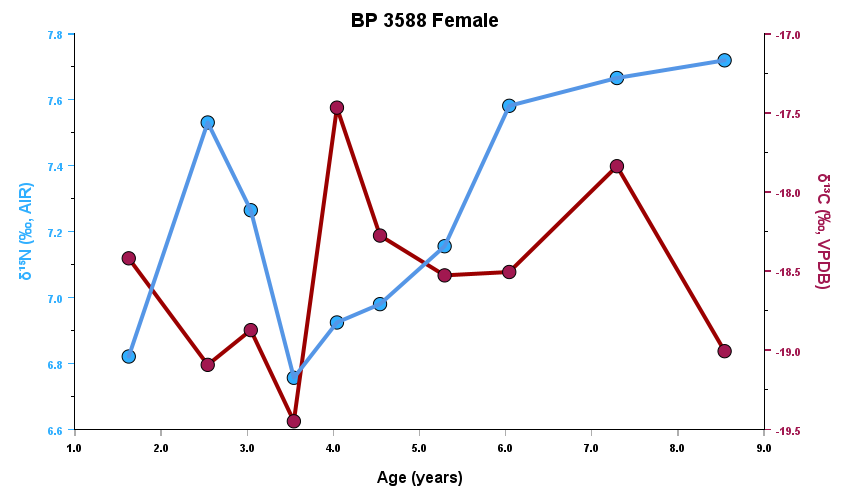

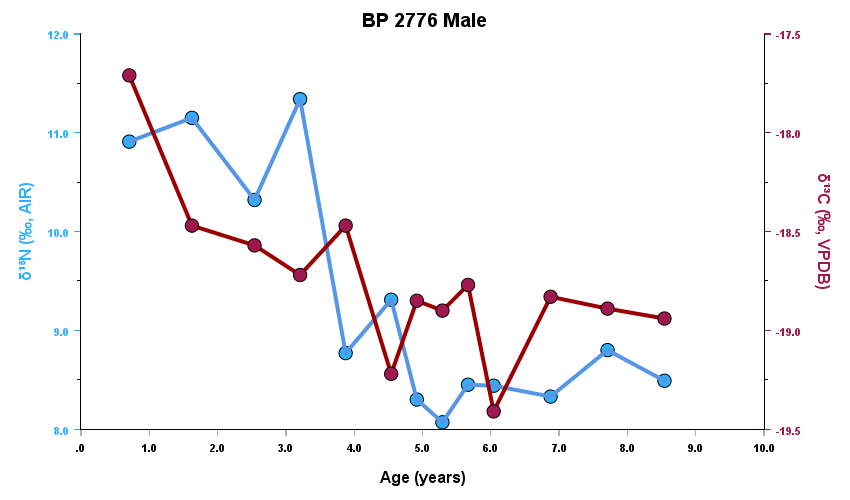

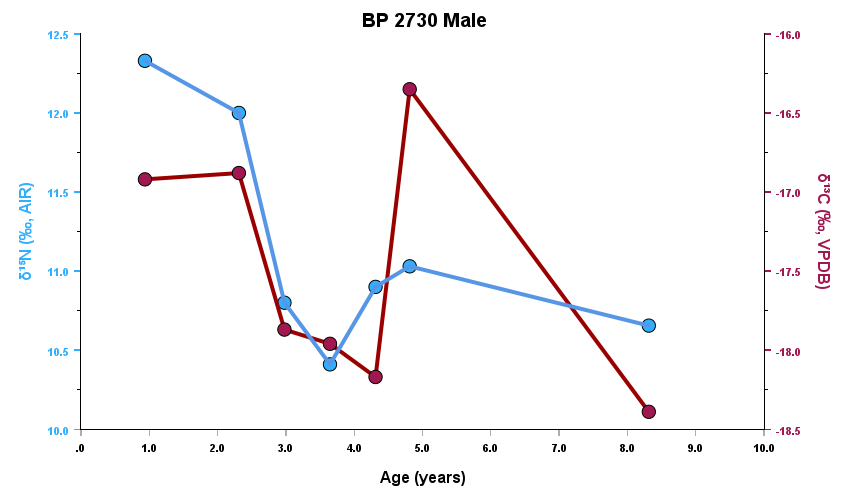


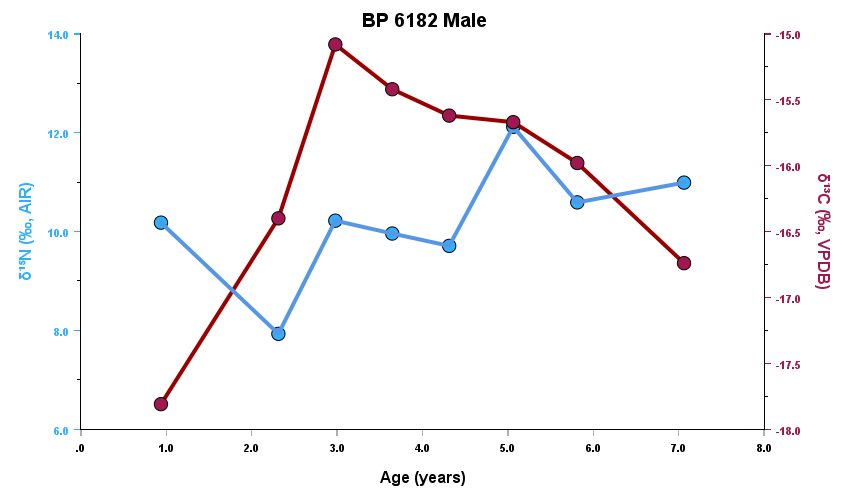

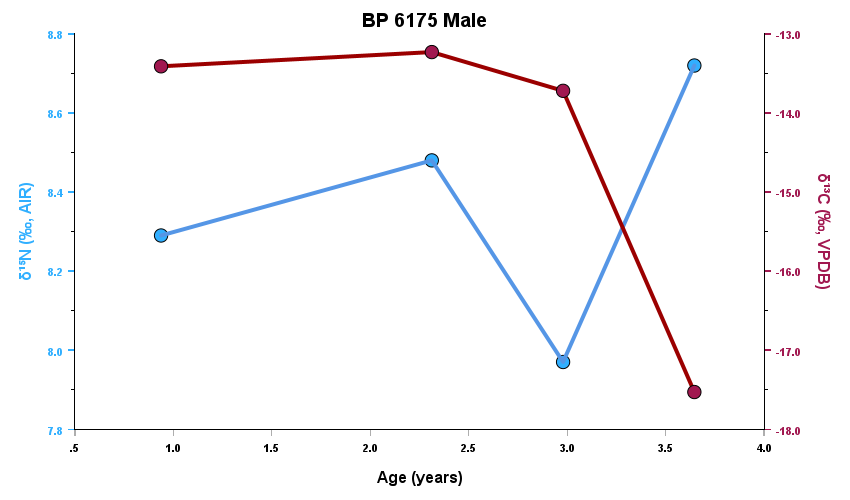

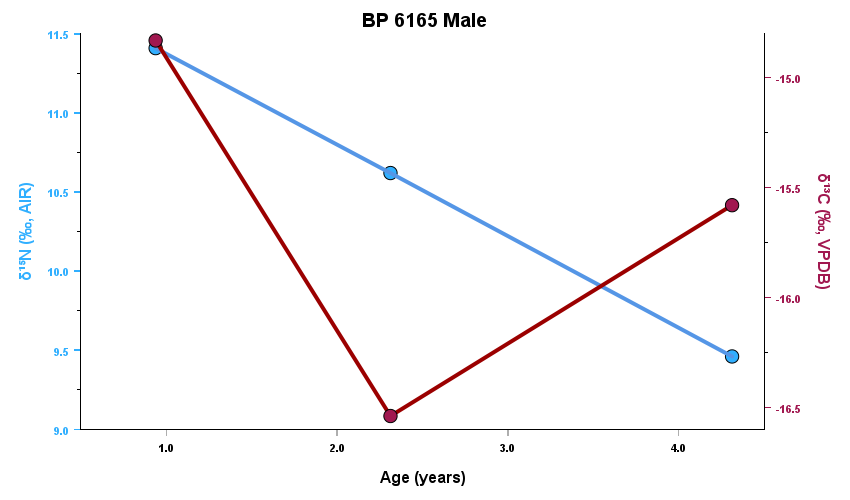

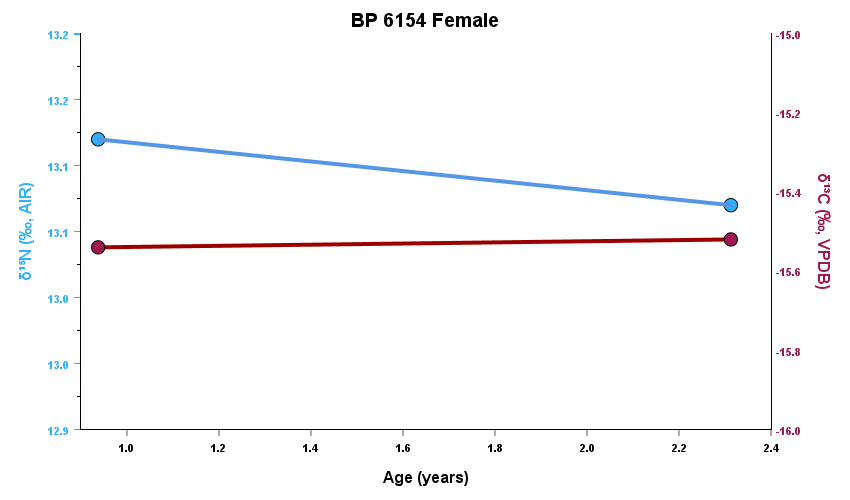


***Montescudaio δ*^13^C and *δ*^15^N profiles**


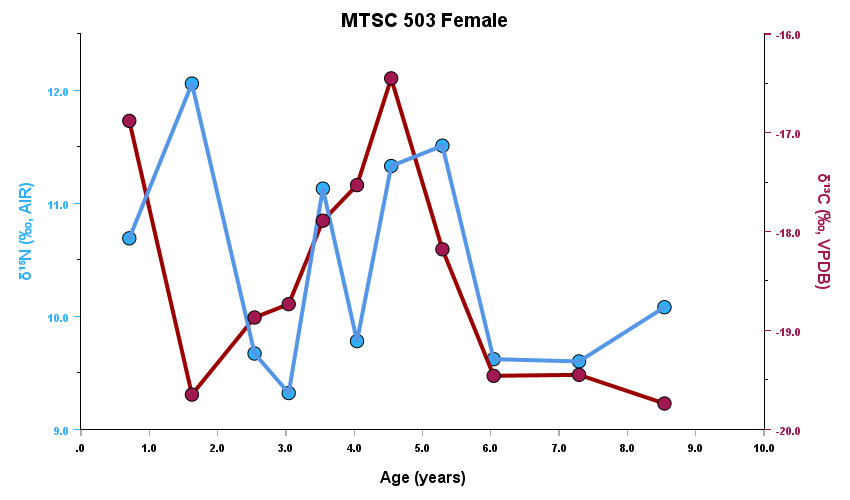

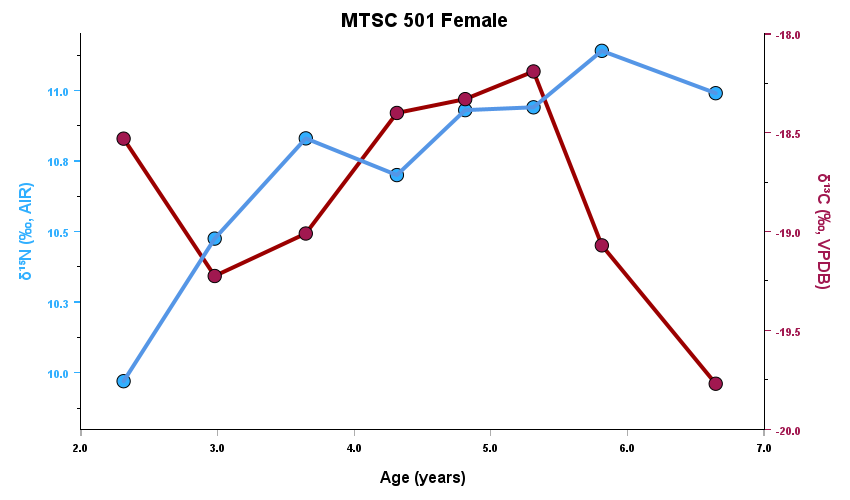

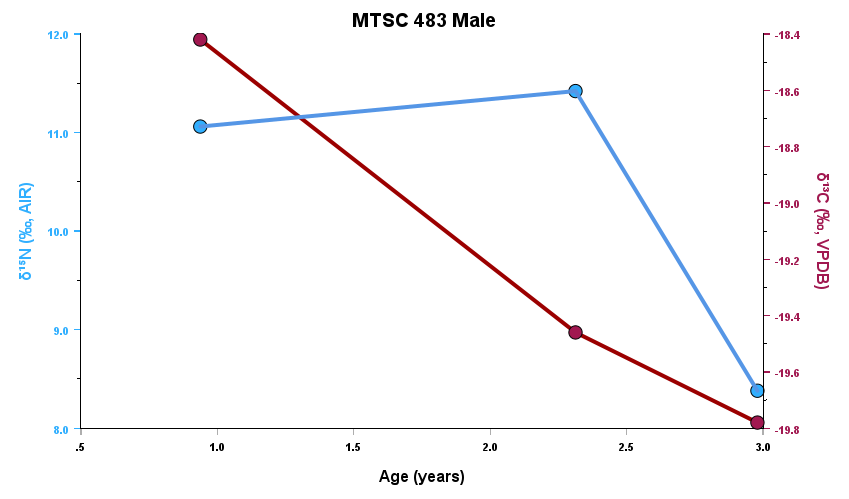

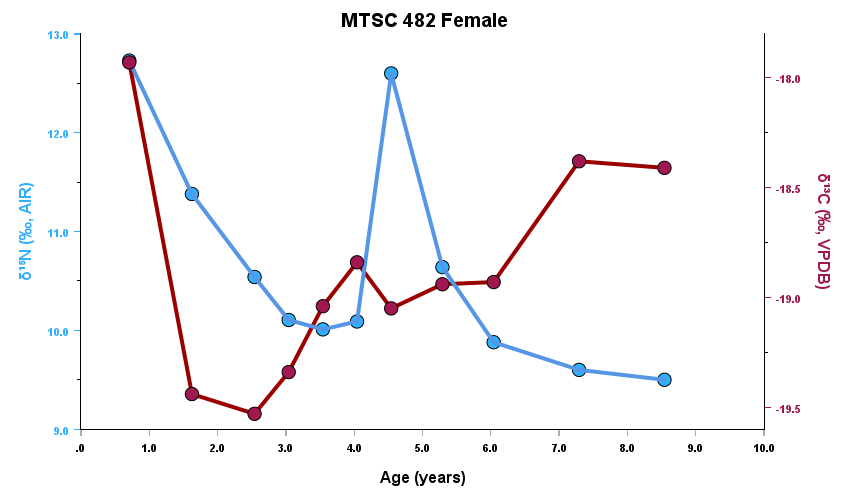

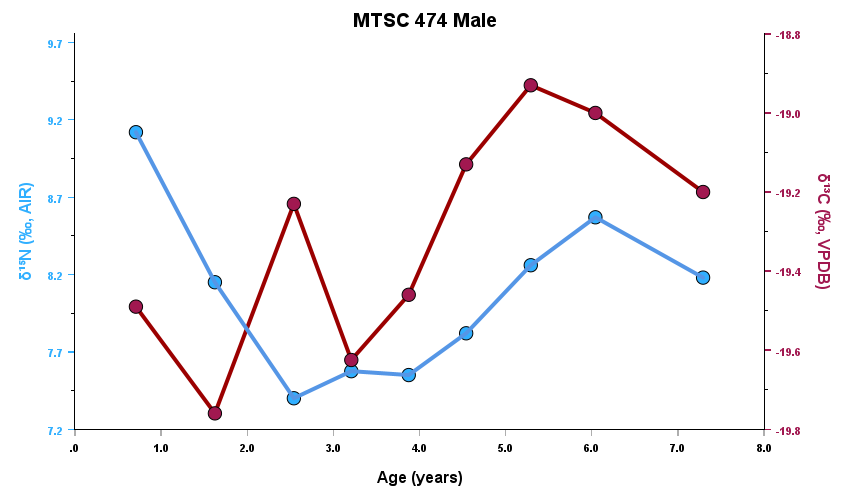

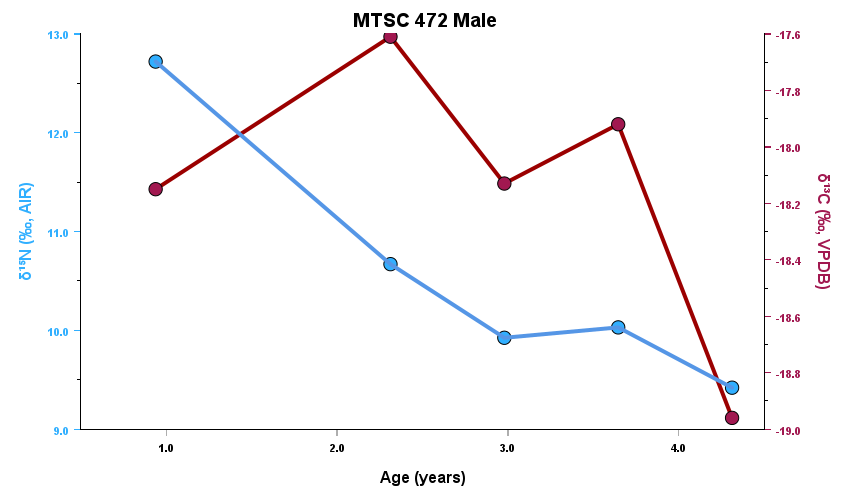

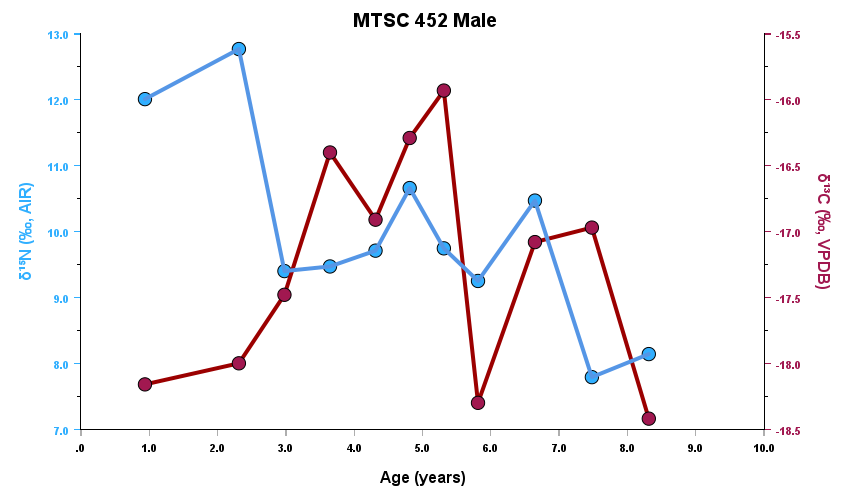

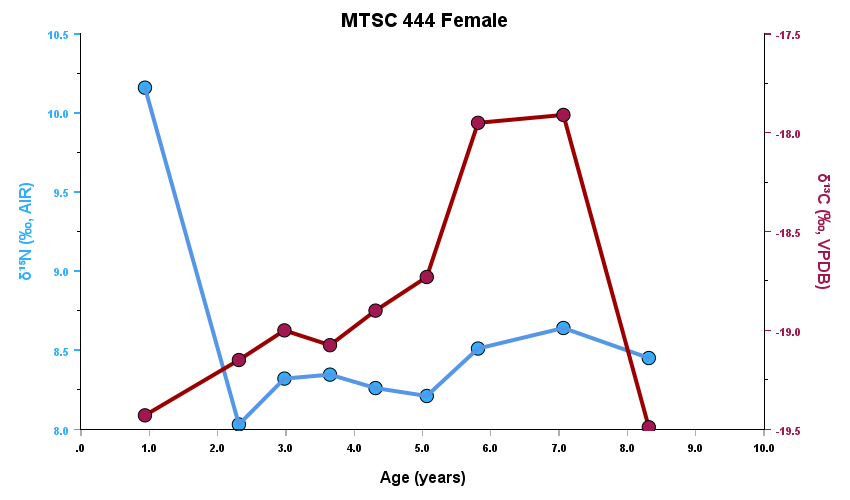

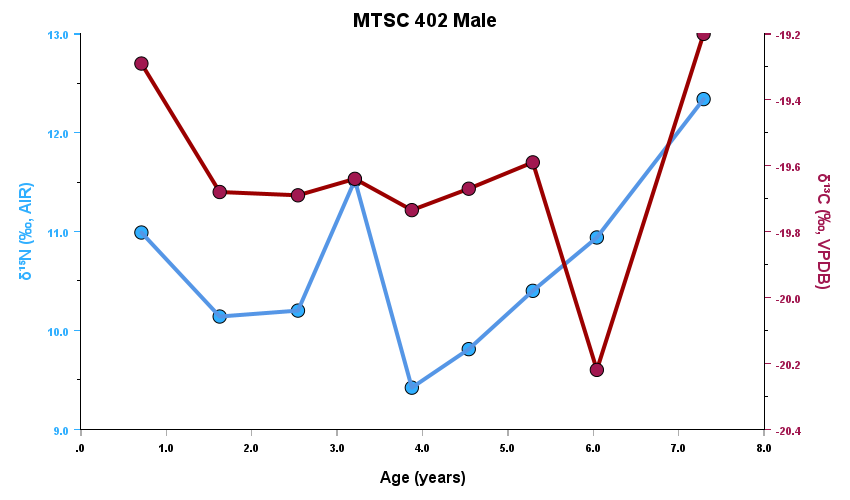

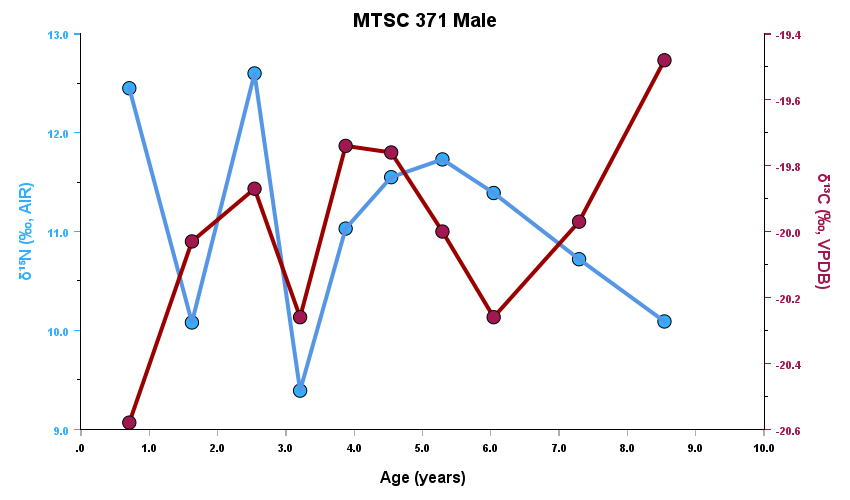

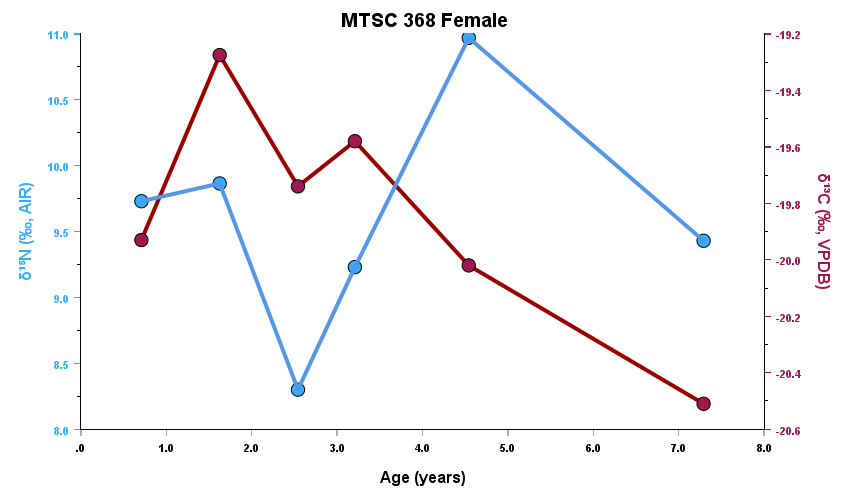

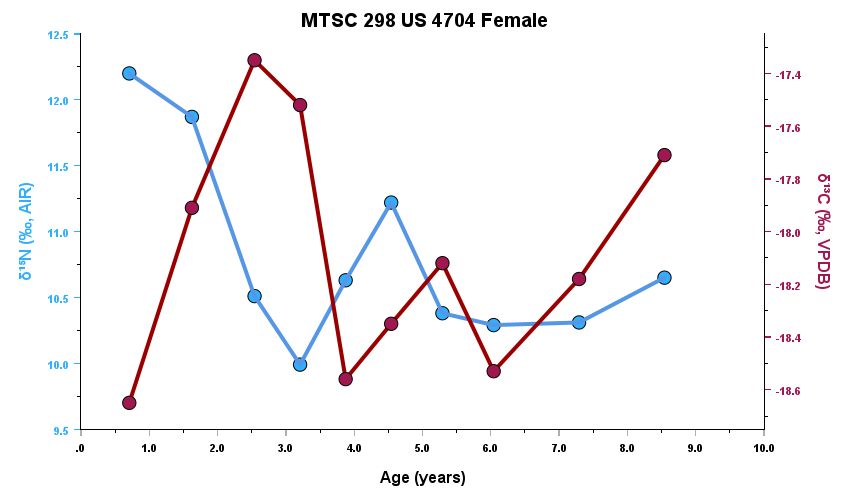

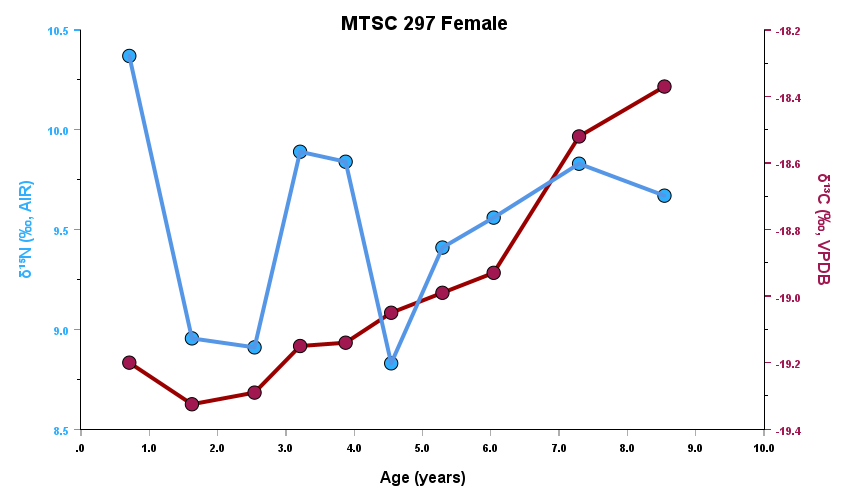

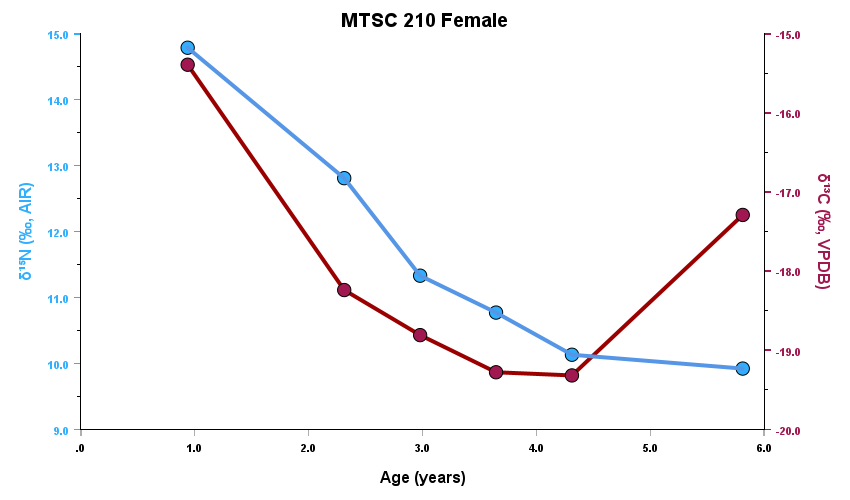

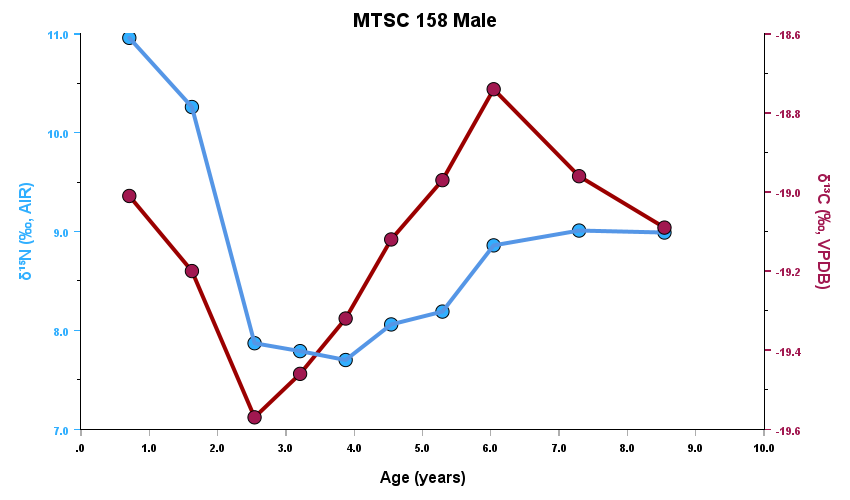

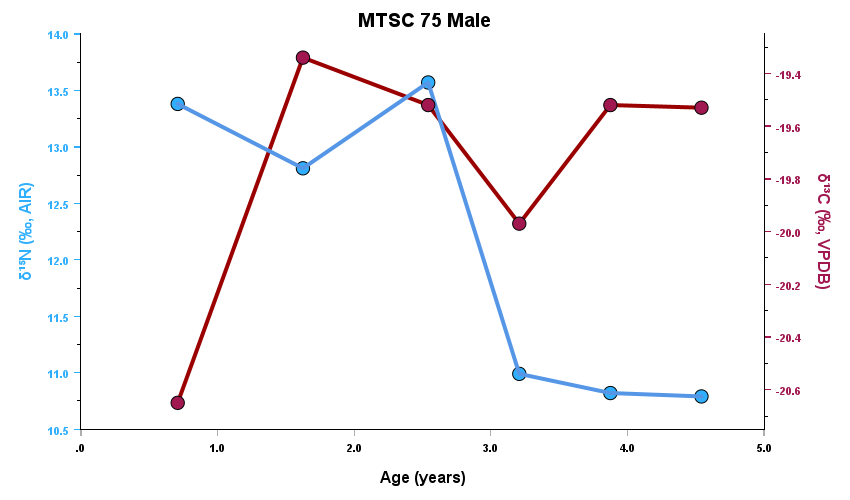

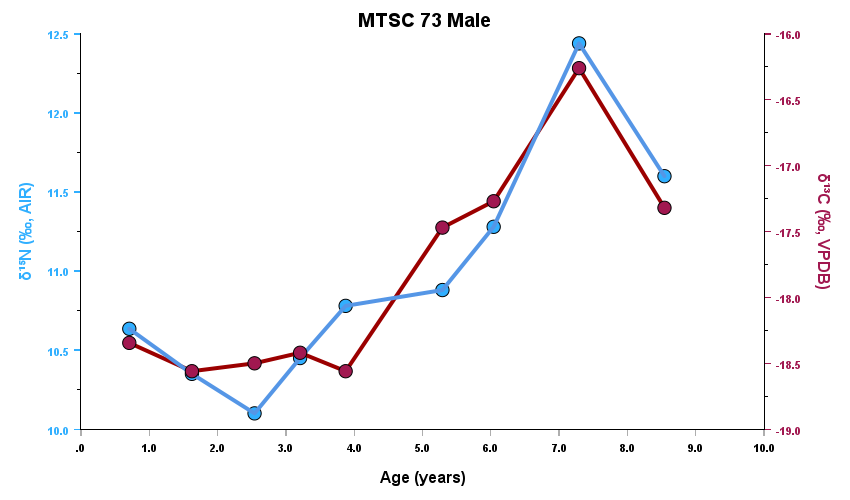

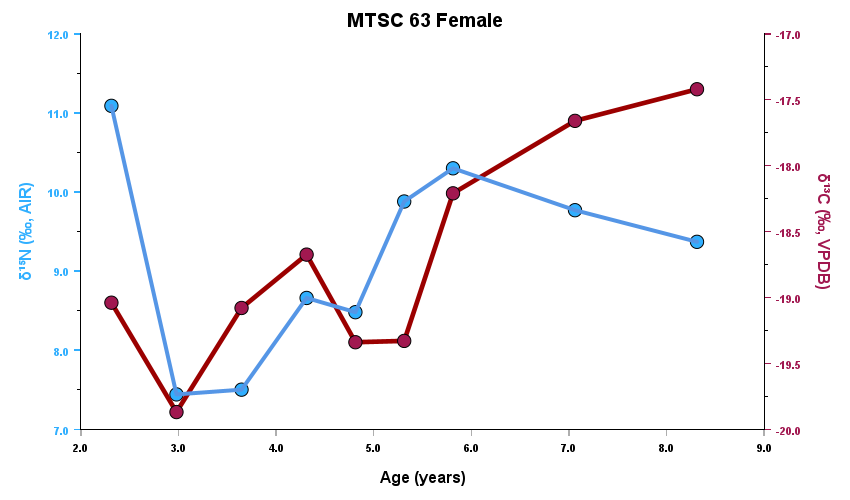

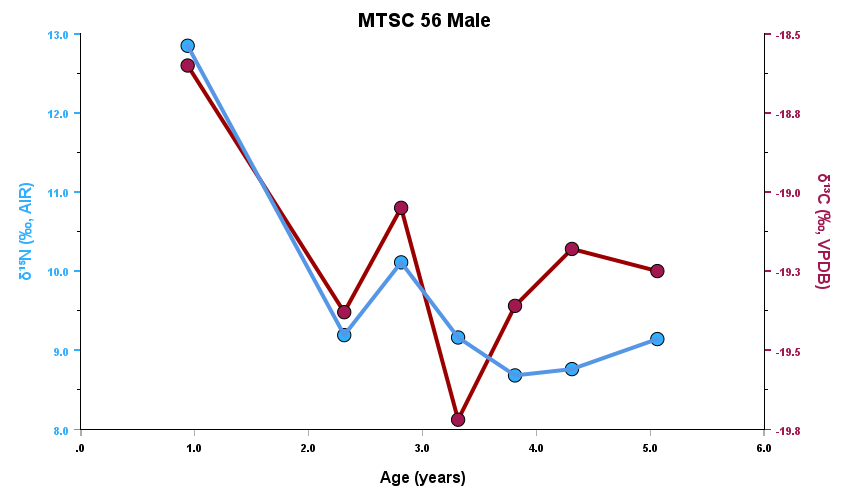

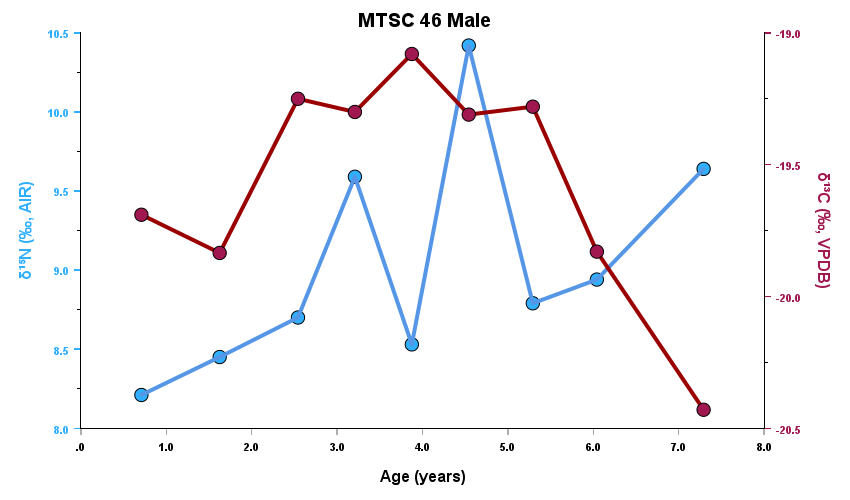

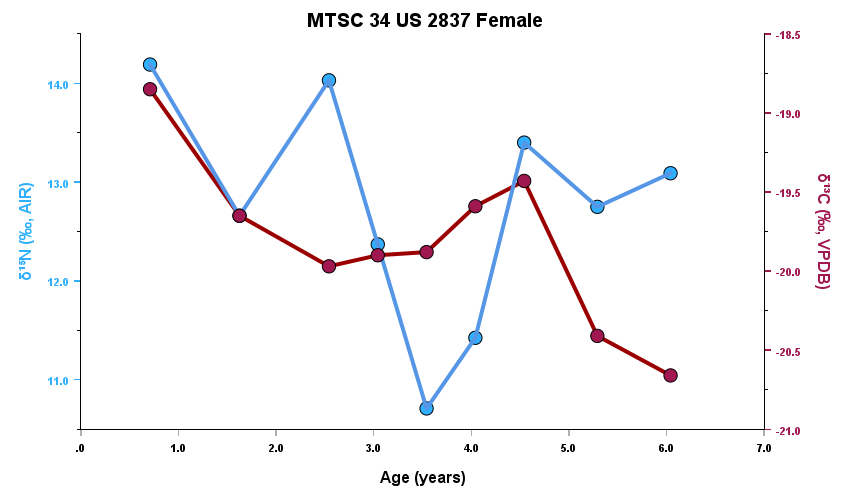

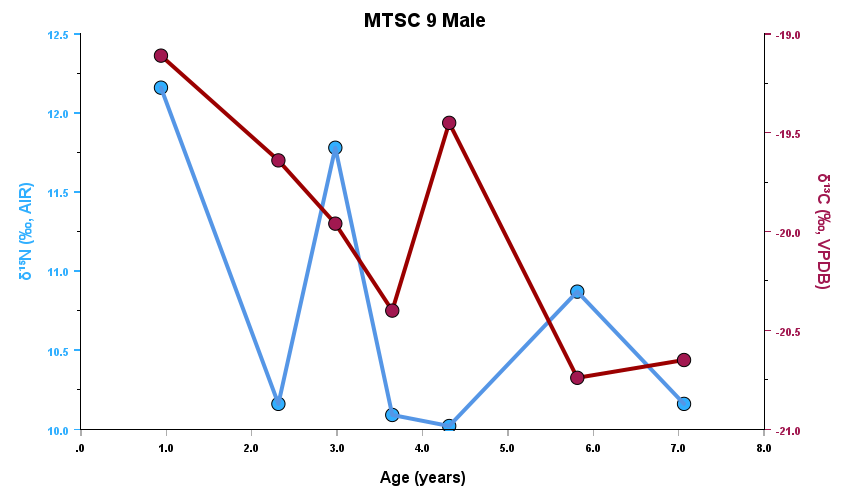

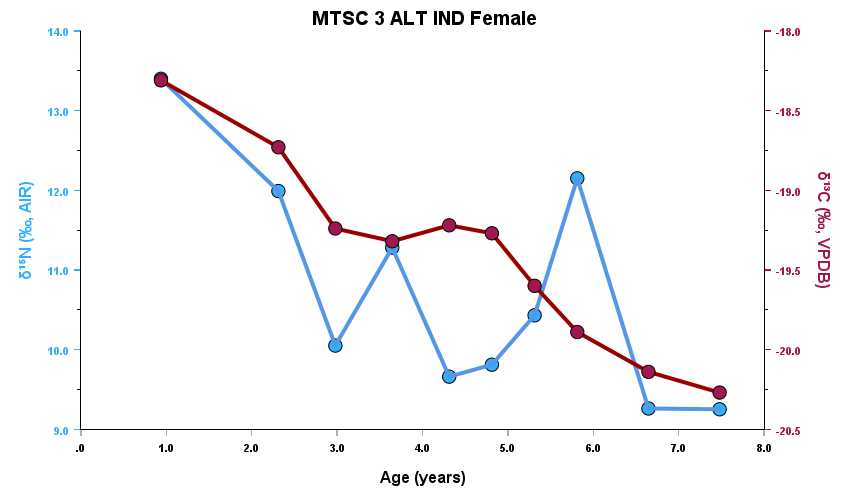

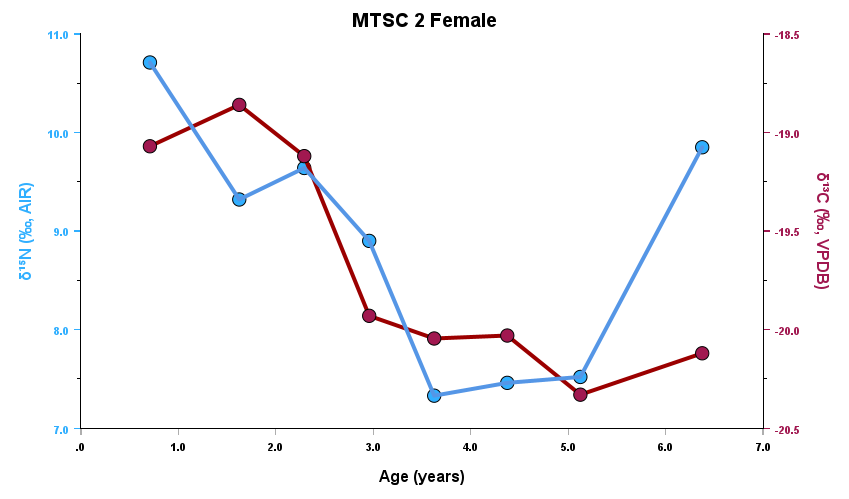


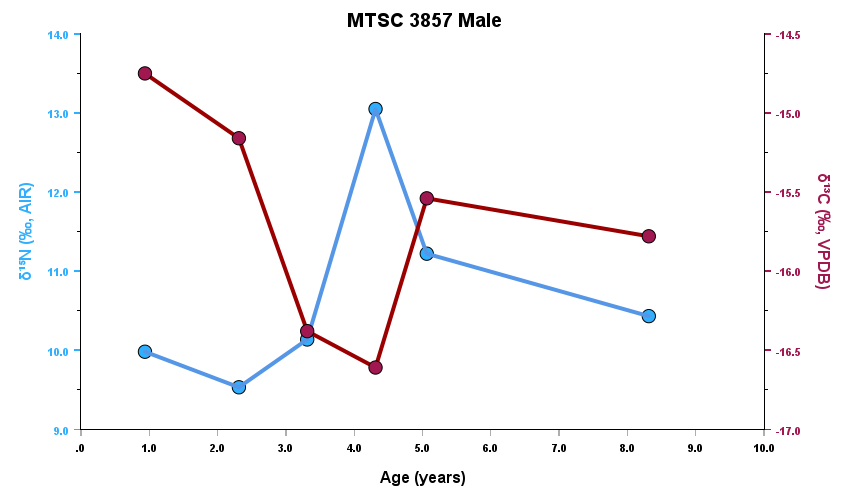

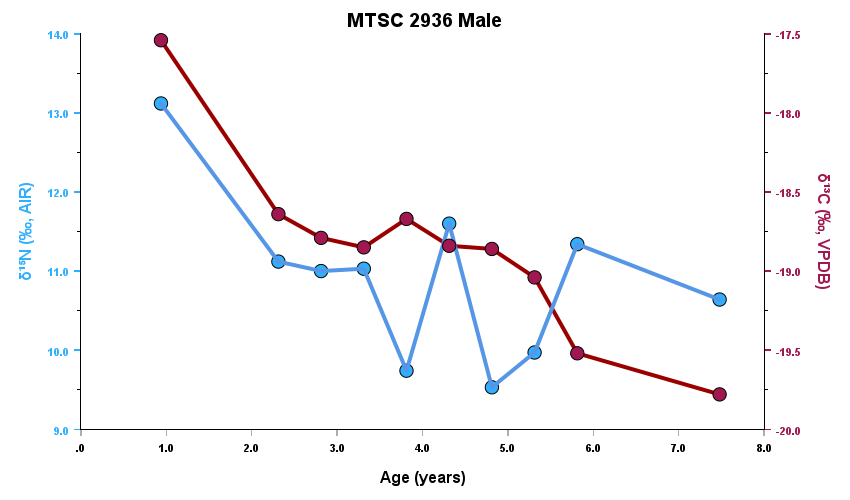

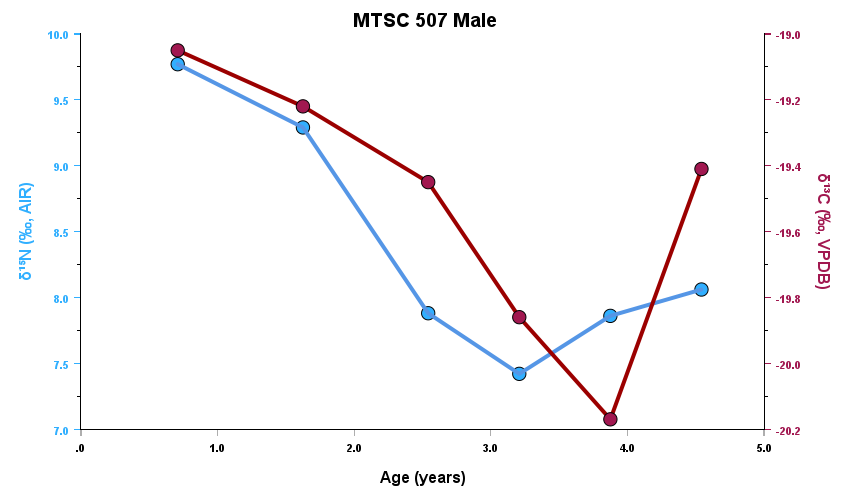

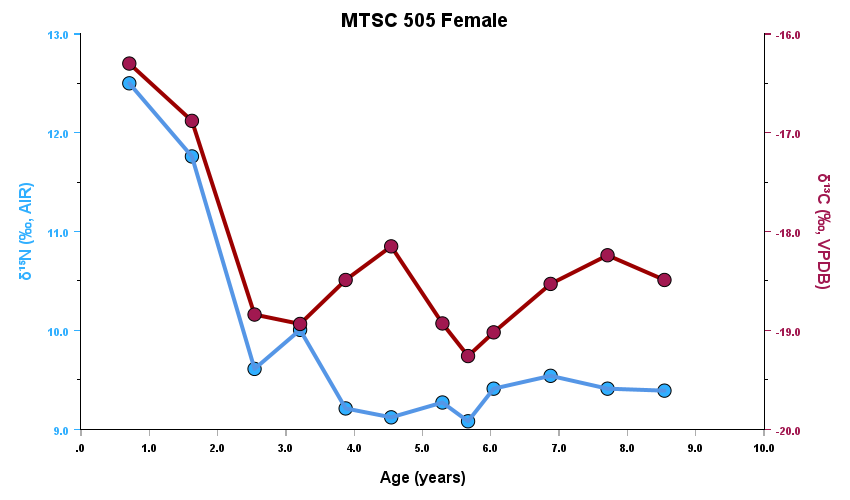


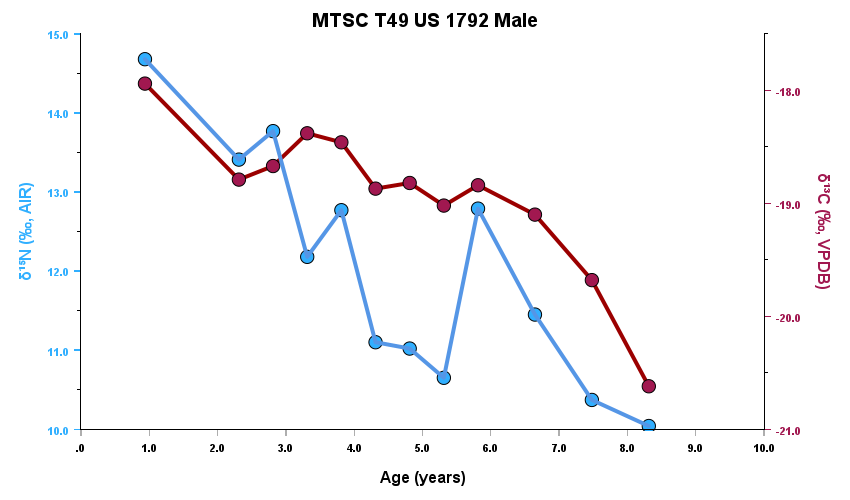

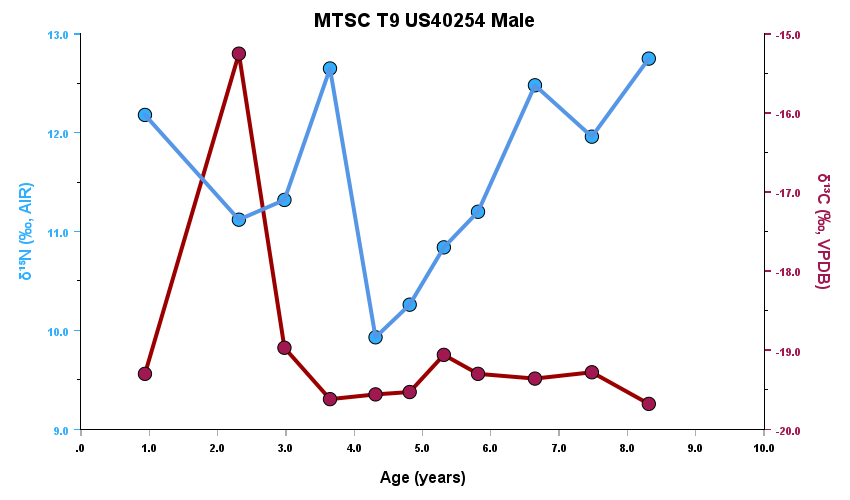

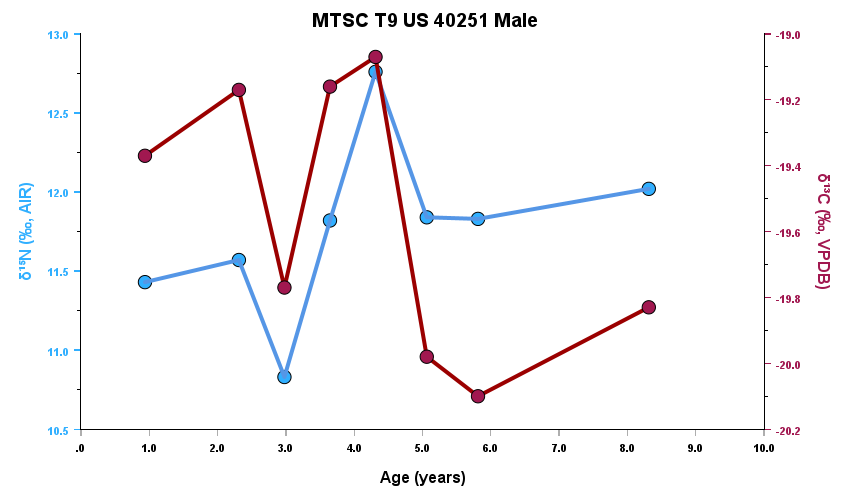

Supplement: S1 File — (DOCX) [file pone.0338595.s001.docx]
